# Recent Advances in Synthesis and Properties of Nitrated-Pyrazoles Based Energetic Compounds

**DOI:** 10.3390/molecules25153475

**Published:** 2020-07-30

**Authors:** Shijie Zhang, Zhenguo Gao, Di Lan, Qian Jia, Ning Liu, Jiaoqiang Zhang, Kaichang Kou

**Affiliations:** 1 MOE Key Laboratory of Material Physics and Chemistry under Extraordinary, School of Chemistry and Chemical Engineering, Northwestern Polytechnical University, Xi’an 710072, China; zsj562389@sina.com (S.Z.); gaozhenguo@mail.nwpu.edu.cn (Z.G.); landi@mail.nwpu.edu.cn (D.L.); qqianjia@163.com (Q.J.); 2 Xi’an Modern Chemistry Institute, Xi’an 710065, China; flackliu@sina.com

**Keywords:** nitrated pyrazoles-based, energetic salts, synthesis, high energy density material, insensitivity

## Abstract

Nitrated-pyrazole-based energetic compounds have attracted wide publicity in the field of energetic materials (EMs) due to their high heat of formation, high density, tailored thermal stability, and detonation performance. Many nitrated-pyrazole-based energetic compounds have been developed to meet the increasing demands of high power, low sensitivity, and eco-friendly environment, and they have good applications in explosives, propellants, and pyrotechnics. Continuous and growing efforts have been committed to promote the rapid development of nitrated-pyrazole-based EMs in the last decade, especially through large amounts of Chinese research. Some of the ultimate aims of nitrated-pyrazole-based materials are to develop potential candidates of castable explosives, explore novel insensitive high energy materials, search for low cost synthesis strategies, high efficiency, and green environmental protection, and further widen the applications of EMs. This review article aims to present the recent processes in the synthesis and physical and explosive performances of the nitrated-pyrazole-based Ems, including monopyrazoles with nitro, bispyrazoles with nitro, nitropyrazolo[4,3-*c*]pyrazoles, and their derivatives, and to comb the development trend of these compounds. This review intends to prompt fresh concepts for designing prominent high-performance nitropyrazole-based EMs.

## 1. Introduction

Energetic materials (EMs), including explosives, propellants, and pyrotechnics, are a significant class of compounds containing large amounts of stored chemical energy, which can liberate heat and exert high pressure under some stimuli, like impact, shock, or thermal effect [1,2,3,4,5,6,7,8]. With the development of science and technology, more and more attention has been paid to the high energy density materials (HEDMs) used for energy and as explosives or propellants [9]. Thus, the representatives of traditional HEDMs are 2,4,6-trinitrotoluene (TNT) [10,11], 1,3,5,7-tetranitro-1,3,5,7-tetrazocine (HMX) [12,13], 1,3,5-trinitro-1,3,5-triazine (RDX) [14], triaminotrinitrobenzene (TATB) [15], and 2,4,6,8,10,12-hexanitro-2,4,6,8,10,12-hexaazaisowurtzitane (CL-20) [16]. The key properties for HEDMs include density (*ρ*), melting point (*T*_m_), decomposition temperature (*T*_d_), heat of formation (HOF), calculated detonation velocity (*D*, calculated propagation velocity of detonation wave in explosive grain), calculated detonation pressure (*P*, calculated pressure on the front of detonation wave), oxygen balance (OB, residual amount of oxygen when explosive explodes to produce CO_2_ and H_2_O, OB = 16[*c-*(2*a* + *b*/2)]/*M* for molecule C_a_H_b_O_c_N_d_), specific impulse (*I*_sp_, impulse produced by the unit quantity of propellant), content of nitrogen (N), impact sensitivity (IS, sensitivity of explosive to impact), friction sensitivity (FS, sensitivity of explosive to friction), electrostatic discharge sensitivity (ESD), and sensitivity of explosive to electrostatic discharge, etc. There are several standards a novel HEDM should meet if it would be applied widely, including insensitivity toward mechanical stimuli (heat, impact, fraction, and electrostatic discharge) to ensure the safety of operation, high performance for various purposes, less toxicity, and producing less hazardous waste after detonation [17,18,19]. Among them, conflict between the increasing energetic level and decreasing sensitivity has become more and more severe. Therefore, the exploration and development of high energy density compounds with low sensitivity have been a priority. A significant amount of effort has been made to resolve this problem, such as recrystallization of Ems [20], preparing polymer bonded explosives (PBXs) [21,22], forming energetic cocrystals [23,24,25], and synthesizing novel energetic compounds [26,27,28,29]. In contrast with other technologies, synthesizing new HEDMs may be the most direct and effective method.

Nitrogen heterocyclic energetic materials that have large numbers of *N*–*N* bonds and *C*–*N* bonds with high energy can form the large π bond similar to benzene, which endows this kind of compounds low sensitivity, high positive heat of formation, and good thermal stability. In addition, the low percentage of C and N in these compounds always lead to high density and good oxygen balance. The decomposition of these compounds can result in the N_2_, which is environmentally friendly [30]. There is a big difference between nitrogen-rich energetic compounds and traditional explosives, namely the energy of nitrogen heterocyclic compounds is released from the high positive heat of formation rather than the oxidation of carbon backbone like traditional explosive (such as TNT and TATB) [19,31]. Therefore, nitrogen heterocyclic materials have garnered large interest in the research areas of HEDMs.

As an outstanding representative of nitrogen heterocyclic compounds, nitropyrazoles and their derivatives are aromatic stable substances with π electrons in their structures. The system is easy to carry out electrophilic substitution reactions such as nitration, sulfonation and halogenation, etc. [32]. These compounds are characterized by oxidation resistance, heat resistance and hydrolysis resistance [19], and are widely applied in civil fields, such as medicine, pesticide, photosensitive materials, and fine chemicals [33,34,35]. Due to the compactness, stability, and modifiability of the molecular structure of pyrazoles, nitration and derivatization of pyrazoles are relatively easy. The ring tension in the structures of nitropyrazoles and their derivatives is large. The density and nitrogen content of nitropyrazoles increase with the presence of nitro groups on the ring, and the oxygen balance is closer to the ideal value, which can improve the detonation performance of the target compounds. Many energetic compounds based on nitropyrazoles have been synthesized successively, which have good applications in highly energy insensitive explosives, propellants, pyrotechnic agents, and other fields [2,3,19,36,37].

In the past decade, a lot of papers on the synthesis and properties of nitrated pyrazoles have been published, including many Chinese references which are not accessible for most Western researchers due to language barriers. This review article presents the recent processes in synthesis, physical and explosive performances of the nitropyrazole-based Ems, including monopyrazoles with nitro, bispyrazoles with nitro, nitropyrazolopyrazoles and their derivatives, and to comb the development trend of these compounds. The aim of this review is to provide readers with an overview of the relationship between structures and properties and guide the future design of novel HEDMs. This review also intends to prompt fresh concepts for designing prominent high-performances nitropyrazole-based EMs.

## 2. Nitrated-Monopyrazole Based Compounds

In this section, the sum of nitro group substituted on carbon position of pyrazole ring in mononitropyrazoles, binitropyrazoles, and trinitropyrazoles are one, two, and three, respectively. For example, mononitropyrazole represents that only one C position in pyrazole ring is substituted by the nitro group.

### 2.1. Mononitropyrazoles and Their Derivatives

Mononitropyrazoles and their derivatives due to their energetic property are favored by people in many fields, such as medicine, pesticide, energetic material and so on. Among them, 3-nitropyrazole (3-NP), 4-nitropyrazole (4-NP), 1-methyl-3-nitropyrazole (3-MNP), and 1-methyl-4-nitropyrazole (4MNP) are typical examples, which are commonly used as energetic materials and intermediates for further products of other energetic materials because they contain only one nitro group and have relatively low energy. The syntheses of these compounds is often facile and can meet the development requirements of green chemistry.

**Figure 1:**
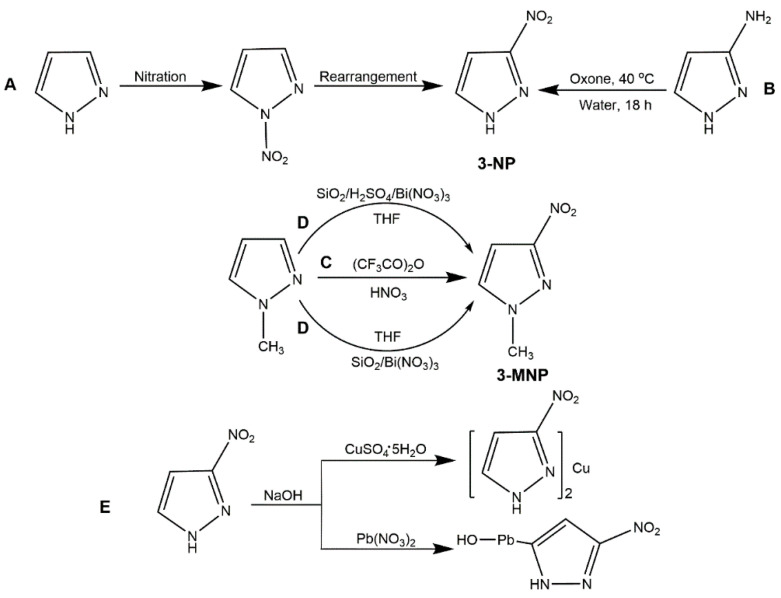
Summary of synthesis of 3-NP, 3-MNP and metal salts of 3-NP.

As a typical heterocyclic compound, 3-NP is an important intermediate in the synthesis of pyrazole-based compounds such as 3,4-dinitropyrazole (DNP) and other new explosives [36,38]. In 1970, Habraken and co-authors [39] firstly reported synthesis of 3-NP by dissolving *N*-nitropyrazole in anisole for 10 h at 145 °C. Later, Verbruggen et al. [40] synthesized 3-NP from diazomethane and chloronitroethylene by one-step cyclization, while this reaction was high riskful due to the extremely vivacious raw materials. Nowadays, the main synthesis method of 3-NP was a two-step reaction, that is, nitration of pyrazole to obtain *N*-nitropyrazole and then rearrangement of *N*-nitropyrazole in organic solvent to acquire 3-NP (Figure 1, Scheme A). The nitration agents could be HNO_3_/H_2_SO_4_ or HNO_3_/Ac_2_O/HAc, and the organic solvent for rearrangement could be anisole, *n*-octanol and benzonitrile [41,42,43]. Among these solvents, benzonitrile was always preferred to be the rearrangement medium since anisole could require an excessively long time and *n*-octanol would lead to poor-quality product. In 2014, Zhao et al. [44] reported one convenient and green approach to synthesizing the 3-NP. They chose the oxone as the nitration agents of 3-aminopyrazole and water as the solvent (Figure 1, Scheme B). This approach owns some advantages over the previous approach: simple operation, safety, economical reagents, the use of water as solvent, and mild conditions. As shown in Figure 1, 3-MNP is one of the most important derivatives of 3-NP. Its synthesis is mainly accomplished by nitrated 1-methylpyrazole with various nitration agents. Katritzky et al. [45] added 1-methylpyrazole to trifluoroacetic anhydride for 1 h in ice bath, and then concentrated nitric acid was added in the solution. After stirring for 12 h, and evaporation of trifluoroacetic anhydride and nitric acid, the 3-MNP could be obtained (Figure 1, Scheme C). In 2013, Ravi et al. [46] proposed that 1-methylpyrazole could reacted with silicon oxide-bismuth nitrate or silicon dioxide-sulfuric acid-bismuth nitrate in tetrahydrofuran (THF) to produce 3-MNP (Figure 1, Scheme D), this facile route is a synthetic method of low toxicity, high efficiency, and green environmental protection. In addition, metal salts of 3-NP expand its derivatives. Li et al. [42] prepared the metal Cu(II) salt and basic Pb salt of 3-NP, by dissolving 3-NP in NaOH solution and reacting with the CuSO_4_·5H_2_O solution and Pb(NO_3_)_2_ solution, respectively (Figure 1, Scheme E).

**Figure 2:**
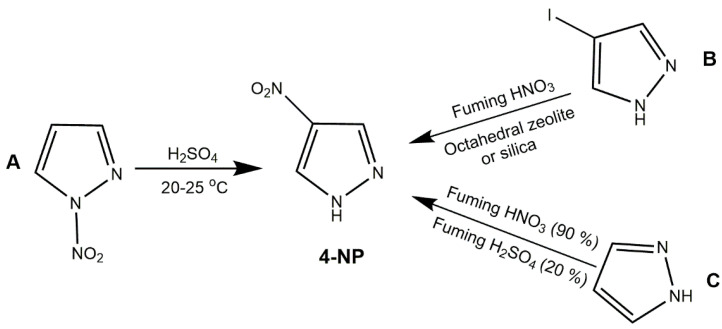
Synthesis of 4-NP.

4-NP is an isomer of 3-NP with melting point of 163–165 °C, density of 1.52 g/cm^3^, detonation velocity of 6.68 km/s and detonation pressure of 18.81 Gpa [47]. Similar to 3-NP, 4-NP can be obtained by nitro group rearrangement. As Rao et al. [48] reported *N*-nitropyrazole could be rearranged to 4-NP in sulfuric acid at room temperature (Figure 2, Scheme A). Ravi et al. [49] synthesized 4-NP in THF with 4-iodopyrazole as raw material, fuming HNO_3_ as nitration agents, octahedral zeolite or silica as solid catalyst (Figure 2, Scheme B). Li et al. [50] reported one-pot two steps route that pyrazole could be nitrated to 4-NP by fuming HNO_3_ (90%)/fuming H_2_SO_4_ (20%) (Figure 2, Scheme C). 4-MNP is another important derivative of nitropyrazole with the similar performance to 3-MNP (Table 1). In 2015, Corte et al. [51] reported that 4-MNP could be synthesized by adding sodium hydride and iodomethane into the THF solution of 4-NP at room temperature for overnight. Ioannidis et al. [52] improved the method by adding sodium hydride and iodomethane to the acetonitrile solution of 4-NP under nitrogen protection for 16 h. However, it is dangerous to handle sodium hydride due to its high chemical reaction activity which can easily cause combustion and explosion, limiting the further application of this method. Han et al. [53] simplified the above method and replaced sodium hydride with potassium carbonate. They added potassium carbonate and iodomethane to the *N*,*N*-dimethylformamide (DMF) solution of 4-NP at 25 °C for 14 h. This method not only reduces the risk in the process, but improves the reaction yield (80–98%).

Table 1 shows the energetic performances of the four typical monopyrazoles. We can see that these energetic performances of pyrazole-based compounds are not satisfying, especially the detonation properties and the nitrogen content. So, these nitropyrazoles are always used as intermediates for the preparation of novel high-performance energetic materials. Furthermore, it is also necessary to explore new high performances energetic materials based on mononitropyrazoles. For example, Deng et al. [54] prepared 5-methyl-4-nitro-1*H*-pyrazol-3(2*H*)-one (MNPO) and its energetic salts, showing better performances than these above mononitropyrazoles.

**Figure 3:**
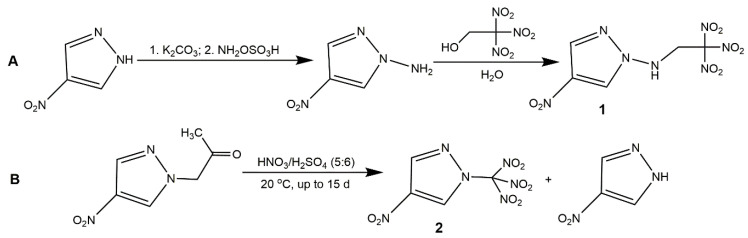
Synthesis of compounds **1** and **2**.

**Figure 4:**
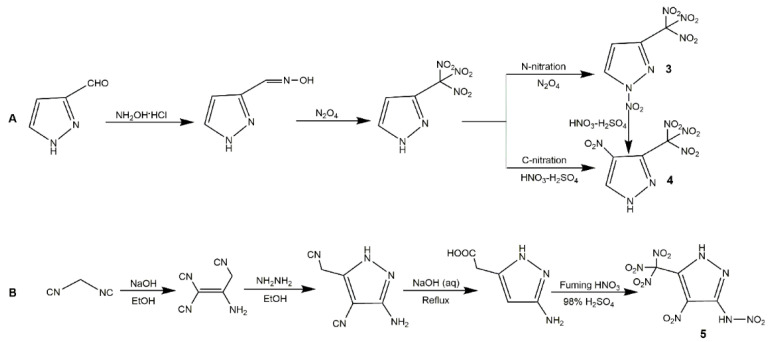
Synthesis of compounds **3**–**5**.

**Figure 5:**
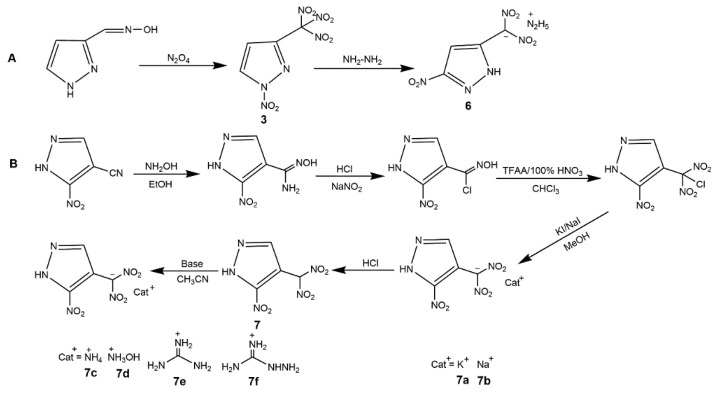
Synthesis of salts of trinitromethyl and dinitromethyl-substituted mononitropyrazoles.

The introduction of a polynitromethyl group into a heterocyclic compound is interesting for energetic field, because it can increase the oxygen content and improve the energetic properties of energetic material. Generally, the incorporation of a polynitromethyl group (trinitromethyl and dinitromethyl) to nitropyrazoles is essentially equivalent to introducing at least one -NO_2_ (since one -NO_2_ is used for the complete oxidation of the C atom in -CH_3_) [56]. For the trinitromethyl group, it can be incorporated into N position or C position of nitropyrazoles with different energetic properties. The N-H bond of nitropyrazole is relatively active which could provide a reaction site for functionalization easily. In 2014, Yin et al. [57] obtained the carbon and nitrogen functionalization of nitropyrazole with *N*-trinitroethylamino group (Figure 3, Scheme A). Thereby, 4-NP reacted with NH_2_OSO_3_H acid and K_2_CO_3_ to accomplish amination, and after functionalization of amino group, the 1-amino-4-nitropyrazole underwent the Mannich reaction with trinitroethanol to get 4-nitro-*N-*(2,2,2-trinitroethyl)-1*H*-pyrazol-1-amine (**1**). In 2015, Dalinger et al. [58] prepared and characterized a nitropyrazole bearing a trinitromethyl moiety at N atom, 4-nitro-1-(trinitromethyl)-pyrazoles (**2**). They synthesized the target compound by a destructive nitration of 4-nitro-1-acetonpyrazole with a mixture of concentrated HNO_3_ and H_2_SO_4_ (Figure 3, Scheme B). Although the compound 1 was successfully synthesized, the yield was very low (28%) and this process was comparatively too time-consuming (15 d). To explore new high-performance EM, several *C*-trinitromethyl-substituted mononitropyrazoles have been reported. In 2018, Zhang and co-authors [56] first synthesized the *C*-trinitromethyl-substituted nitropyrazole (Figure 4, Scheme A). The reaction of 3-pyrazolecarbaldehyde oxime with N_2_O_4_ produced the 3-trinitromethylpyrazole and 1-nitro-3-trinitromethylpyrazole (**3**). They found that the increasing N_2_O_4_ concentration could improve the proportion of **3** and 3-trinitromethylpyrazole reacting with N_2_O_4_ also form **3**, indicating N_2_O_4_ enable nitrate the N position of pyrazole. After the introduction of trinitromethyl group on C position, the 4-nitro-3-trinitromethylpyrazole (**4**) could be obtained with fuming nitric acid and oleum by -NO_2_ rearrangement of **3** or nitration of 3-trinitromethylpyrazole. In 2019, Xiong et al. [59] further designed 3-Trinitromethyl-4-nitro-5-nitramine-1*H*-pyrazole (**5**). It was notable that the yield of **5** could improve with the concentration of HNO_3_ increasing in the last nitration step of Scheme B (Figure 4). For the dinitromethyl group, Semenov et al. [60] prepared the 4-nitro-1-dinitromethylpyrazole by nitrating 4-nitro-1-acetonylpyrazole using H_2_SO_4_/H_2_O mixture, and while the yield was low and it was not investigated as energetic material. In 2019, Pang et al. [61] introduced the dinitromethyl group into nitropyrazole and developed the salt, hydrazinium 5-nitro-3-dinitromethyl-2*H*-pyrazole (**6**), according to Scheme A in Figure 5. In 2020, Cheng et al. [62] synthesized 3-nitro-4-dinitromethyl-2*H*-pyrazole (**7**) and its salts, further exploring the application of dinitromethyl group in mononitropyrazolle. Table 2 shows the energetic properties of the polynitromethyl-substituted mononitropyrazoles and salts compared with TNT and RDX. All the density of the derivatives of mononitropyrazole was higher than TNT and close to that of RDX, especially **7a** showed the highest density. **3** and **5** owned the desirable detonation properties, while exhibited poor safety. It was notable that *C*-trinitromethyl-substituted derivatives owned higher heat of formation than those of *N*-trinitromethyl-substituted derivatives, and the derivatives with dinitromethyl group owned lower heat of formation than derivatives with trinitromethyl group. Most of the neutral derivatives hold low decomposition temperatures owing to the instability of the polynitromethyl moiety. Compound **4** had the highest decomposition temperature possibly because of the strong intermolecular hydrogen bonding interactions. By comparing **4** and **5**, we can see the nitramino group could further increase the power with low sensitivities. For the salts of compound **7**, **7d** with high detonation properties (comparing with RDX) and low sensitivities could serve as a promising candidate as a new high energy density oxidizer.

**Figure 6:**
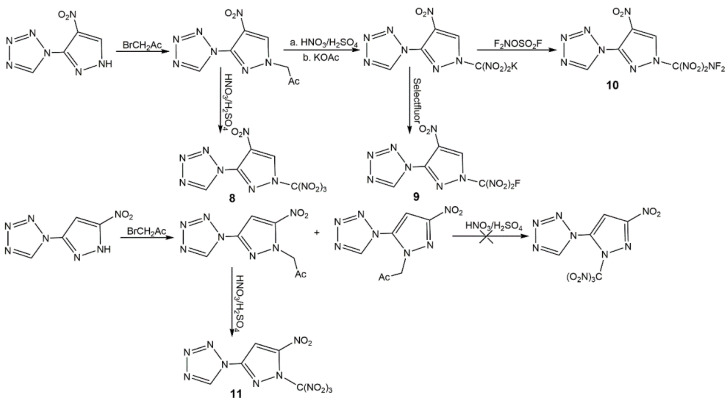
Synthesis of mononitropyrazole derivatives **8**–**11**.

**Figure 7:**
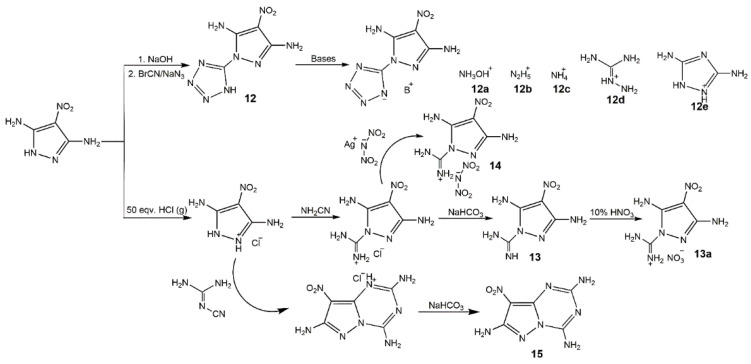
Synthesis of mononitropyrazole derivatives **12**–**15**.

Connecting nitropyrazoles with nitrogen-rich compounds (including tetrazole, triazole, furazan, tetrazine, triazine, and others) has attracted more interest in many fields, it also be an effective approach to increasing the content of nitrogen and getting new high-performance energetic materials. In 2015, Yin et al. [63] synthesized energetic salts based on *N*-methyl 6-nitropyrazolo[3,4-*d*][1,2,3]triazol-3(4*H*)-olate in a similar manner exhibiting good detonation performance with relatively low sensitivities. In 2016, Dalinger et al. [64] synthesized and investigated systematically a series of 1- and 5-(pyrazolyl)tetrazole amino and nitro derivatives which could be components of dyes and luminophores, and high-energy materials. Some of them were always used as intermediates due to their poor energetic properties. In 2017, Zyuzin et al. [65] introduced the 2,2-bis(methoxy-*NNO*-azoxy)ethyl group to nitropyrazoles to increase the hydrogen content for some special application (gun propellants, solid rocket propellants and others). The derivatives of 3-NP and 4-NP showed high heat of formation, while the oxygen balances and calculated detonation velocity were not ideal. Then, Zyuzin et al. [66] further introduced the trinitromethyl moiety owning the most oxygen-rich block into the combination of tetrazole and pyrazole rings to obtain oxygen-balanced energetic materials with high nitrogen content (**8**–**11**) (Figure 6). In 2019, Tang et al. [67] developed several compounds and salts based 3,5-diamino-4-nitropyrazole functionalizing the with tetrazole group and triazine group (**12**–**15**) (Figure 7). As shown in Table 3, all the compounds had high density, high nitrogen content and good detonation properties, while the thermal stability of **12**–**15** was better than that of **8**–**11**. In particular, the derivatives **12**–**15** showed excellent insensitivities. In addition, most compounds owned positive and high heat of formation, but the presence of water molecules in **13a** result in its negative heat of formation. Considering the low sensitivities, good detonation properties, and high thermal stabilities, these derivatives with nitrogen-rich groups may be the candidates of insensitive high energetic materials.

**Figure 8:**
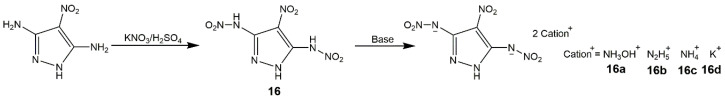
Synthesis of compound **16** and its salts.

**Figure 9:**
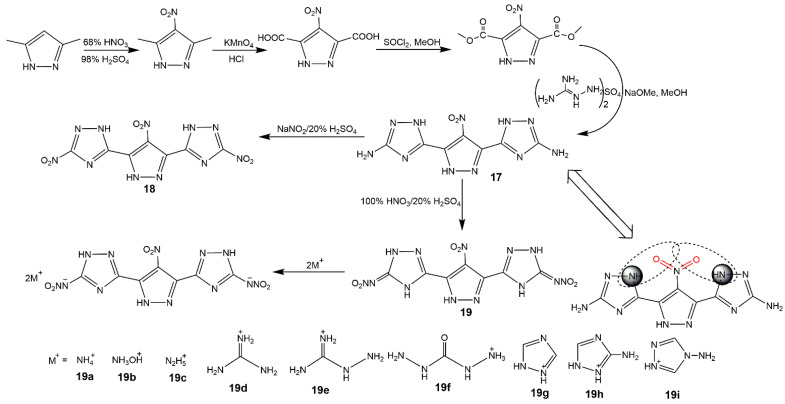
Synthesis of compounds **17**–**19i**.

**Figure 10:**
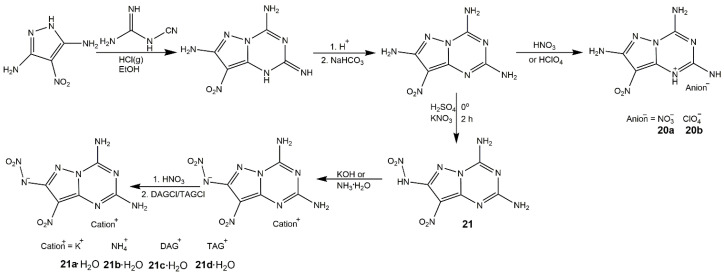
Synthesis of compounds **20a**–**21d**.

Moreover, nitrogen-rich heterocycles with a nitramino moiety could exhibit better performance than the corresponding nitro-substituted analogs as above mentioned [59,68]. In 2019, Shreeve and her group [69] reported a green synthetic route for high-performance nitramino nitropyrazoles. Figure 8 depicted the synthesis of corresponding derivatives, among them the 3,5-dinitramino-4-nitropyrazole (**16**) was quite sensitive to mechanical stimulation. From Table 4, the compound **16b** showed promising properties with a high density (1.87 g·cm^−3^), good detonation properties (*D* of 9.58 km·s^−1^ and *P* of 38.5 GPa), decomposition temperature of 194 °C, and acceptable sensitivities. Xu et al. [70] introduced nitramino and triazole groups into mononitropyrazole to construct multiple hydrogen bonds (**17**), and synthesized the 4-nitro-3,5-bis(1*H*-1,2,4-triazol-3-nitramino)-1*H*-pyrazole (**19**) and its ionic derivatives (**19a**–**i**) as shown in Figure 9. Table 4 also showed their energetic properties. Compound **17** had the highest decomposition temperature (353.6 °C) and excellent low sensitivity (IS > 40, FS > 360), indicating it could be used as heat-resistant insensitive explosive. The compounds (**18**–**19i**) exhibited moderate detonation properties, high positive heat of formation and ideal insensitivities which had great potential application in green and safe energetic materials. Ma et al. [71] also fused nitropyrazole with triazine and nitramino groups, and prepared a series of salts based on compounds **20** and **21** (Figure 10). These compounds owned high thermal stability and excellent insensitive properties because of the existence of triazine ring.

In summary, most of mononitropyrazoles and their derivatives owned relatively low thermal properties and detonation properties. They are always used as intermediates for novel complicated energetic materials. The introduction of polynitromethyl group can improve the oxygen balance efficiently, while have a little influence on the heats of formation. The nitramino group and nitrogen-rich heterocyclic can enhance the detonation properties, improve the safety, and increase the heats of formation of mononitropyrazoles. The choice of solvent and nitrification in synthesis routes should be more environmental and facile.

### 2.2. Dinitropyrazoles and Their Derivatives

Dinitropyrazoles own higher density and better detonation performance than mononitropyrazoles attributing to one more nitro group. The typical dinitropyrazoles include 3,4-dinitropyrazole (3,4-DNP), 3,5-dinitropyrazole (3,5-DNP), 1-methyl-3,4-dinitropyrazole (3,4-MDNP), 1-methyl-3,5-dinitropyrazole (3,5-MDNP), and 4-amino-3,5-dinitropyrazole (LLM-116).

**Figure 11:**
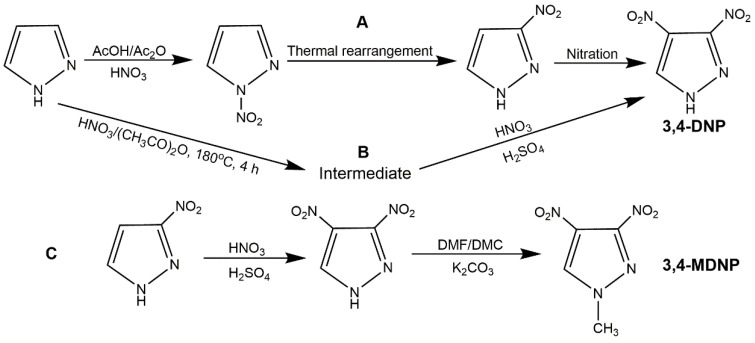
Synthesis of 3,4-DNP and 3,4-MDNP.

**Figure 12:**
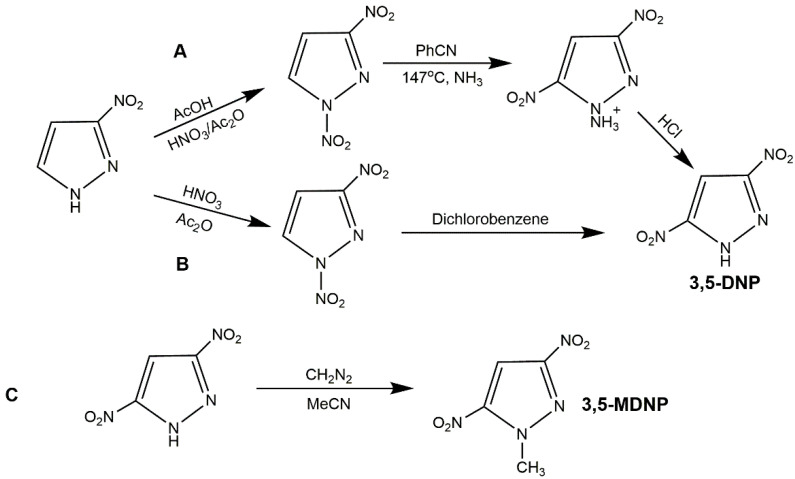
Synthesis of 3,5-DNP and 3,5-MDNP.

3,4-DNP is a kind of white crystal, possessing higher density (1.87 g·cm^−3^), lower melting point (86–88 °C), higher decomposition temperature (285 °C), higher detonation velocity (8.1 km·s^−1^) and detonation pressure (29.4 GPa) than TNT. This compound was first reported by Biffin’s team in 1966 [72]. In an earlier study, pyrazole, 4-NP, 3-nitro-4-cyanopyrazole and other raw materials have been investigated to prepare 3,4-DNP, while most of the methods did not satisfied industrialization due to complex process, high production cost or low yield [45,55,73,74,75,76]. At present, the three-step synthetic route as shown in Figure 11 (Scheme A), and the two-step route (Scheme B) are the most widely used [77,78,79,80]. 3,4-MDNP is a typical thermal stability nitropyrazole, exhibiting stable thermodynamic state at 300 °C. Its melting point and density are lower than those of 3,4-DNP (20–23 °C, 1.67 g·cm^−3^), and 3,4-DNP shows low detonation velocity (7.76 km s^−1^) and detonation pressure (25.57 GPa) due to the introduction of methyl group. It has potential application in liquid explosive, which can reduce the melting point of liquid phase carrier in castable explosive [32]. Recently, Ravi et al. [73] had synthesized 3,4-MDNP by nitrating 1-methylpyrazole or 1-methyl-3-nitropyrazole with montmorillonite (K-10) and Bi(NO_3_)_3_, while this method was high cost and the products were difficult to separate. Li et al. [81] reacted 3,4-DNP and dimethyl carbonate (DMC) in DMF with K_2_CO_3_ as catalyst, then, his group further synthesized 3,4-MDPN with 3-NP as raw material (Figure 11, Scheme C) [82]. In this method, DMC was used as methylation agent and the yield of methylation was high (95.6%), which could meet the requirement of green chemistry. As 3,5-DNP with a melting point of 173–174 °C and density of 1.80 g·cm^−3^, the decomposition temperature of 316.8 °C owns higher detonation properties than 3,4-DNP (7.76 km·^−1^ and 25.57 GPa). Moreover, 3,5-DNP is relatively stable because of the symmetrical molecular distribution, it can be used as a simple explosive or as a key intermediate in the synthesis of insensitive explosives [55]. Generally, the starting materials for preparing 3,5-DNP could be pyrazole and 3-NP. Wang et al. [83] nitrated 3-NP to get 1,3-dinitropyrazole, then 1,3-dinitropyrazole was reacted with NH_3_ in PhCN to produce the ammonium salt of 3,5-DNP. After neutralization with hydrochloric acid, the 3,5-DNP could be obtained (Figure 12, Scheme A). Liu et al. [28] also nitrated 3-NP, and rearranged 1,3-dinitropyrazole to get 3,5-DNP (Figure 12, Scheme B). For pyrazole as starting material, 3,5-DNP was always prepared by a four-step route (nitration of pyrazole, rearrangement of *N*-nitropyrazole, nitration of 3-NP, and rearrangement of 1,3-dinitropyrazole). 3,5-MDNP owns the similar energetic properties with 3,4-MDNP, while it has a higher melting point (about 60 °C). Moreover, 3,5-MDNP could be synthesized by methylation of 3,5-DNP [84]. However, most methylation agents were extremely toxic, thus searching for a green methylation agent would be the key factor.

**Figure 13:**
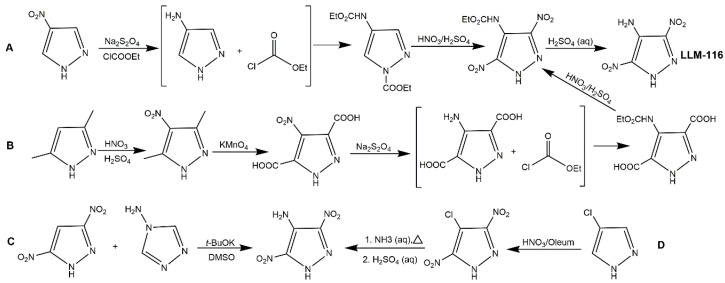
Synthesis of LLM-116.

LLM-116 is a powerful and insensitive explosive, its energy is 90% of HMX and its impact sensitivity is extremely low [55,85]. It was first synthesized by the Lawrence Livermore National Laboratory (LLNL) in 2001, and many studies were performed to assess its synthesis in the following years. Wang et al. [86] utilized vicarious nucleophilic substitution (VNS) of 3,5-DNP and trimethylhydrazine iodideto (TMHI) to prepare LLM-116 with a yield of 60%, while the toxic TMHI was the main factors restricting wide application of this method. In 2014, Stefan et al. [87] developed four synthetic routes of LLM-116, using 4-NP, 3,5-dimethylpyrazole, 3,5-DNP and 4-chloropyrazole as starting materials, respectively (Figure 13, Scheme A–D). Table 5 shows the comparison of the four routes. The synthesis of Scheme D was simple and its yield was high, which was suitable for industrialization. Zhang et al. [88] also used 4-chloropyrazole as a starting material to synthesize LLM-116 with an overall yield of 65%.

**Figure 14:**
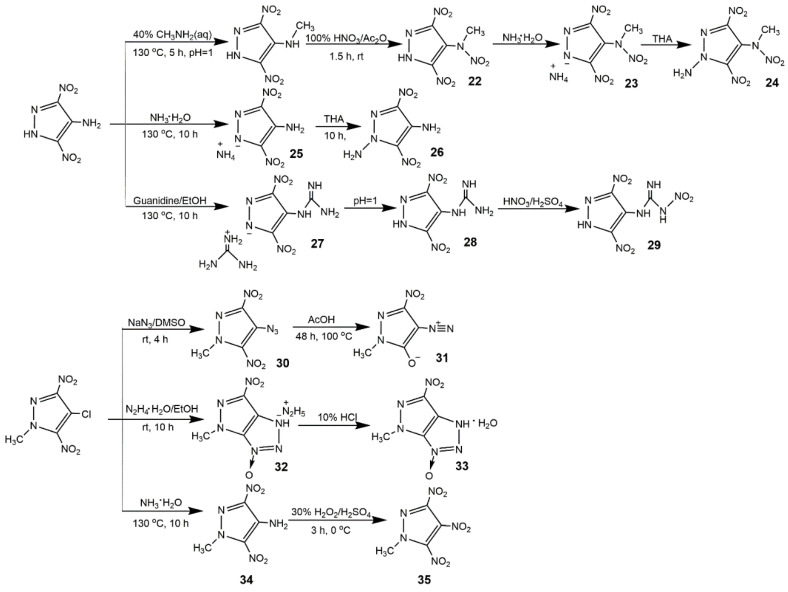
Synthesis of derivatives based on 4-amino-3,5-dinitropyrazole and 1-methyl-4-chloro-3,5-dinitropyrazole.

In addition, 4-Chloro-3,5-dinitropyrazole was a useful intermediate in the preparation of various 3,5-DNP [89], owning good reactivity towards nucleophiles. He et al. [90] synthesized a series of 3,5-DNP derivatives based on 4-chloro-3,5-dinitropyrazole and 1-methyl-4-chloro-3,5-dinitropyrazole shown in Figure 14. From Table 6, all compounds exhibited better detonation properties than those of TNT, and these compounds owned better IS than RDX except compound **33**. Compounds **26** and **28** had an especially good balance between physical properties and detonation properties as well as excellent insensitivity, making them potential replacement of RDX.

**Figure 15:**
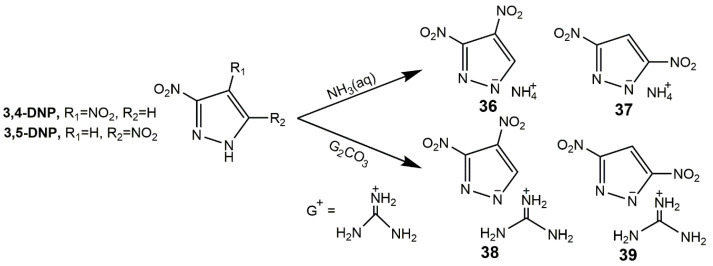
Synthesis of ionic salts of 3,4-DNP and 3,5-DNP.

**Figure 16:**
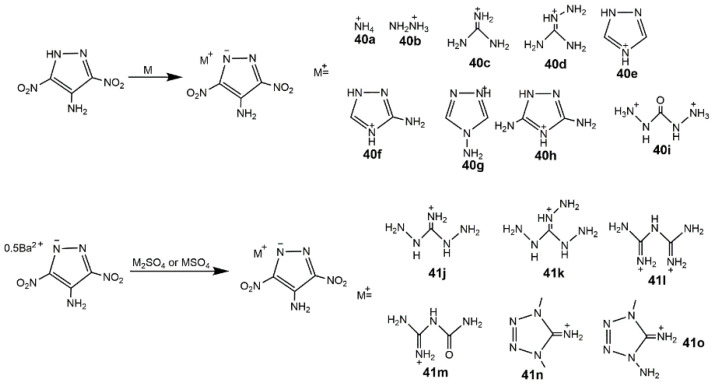
Synthesis of ionic salts of LLM-116.

Energetic salts often possess superior properties comparing with non-ionic species since they always show lower vapor pressures, lower impact and friction sensitivities, and enhanced thermal stabilities [19]. In addition to the derivatives mentioned above, Klapötke group [26] developed the ionic salts of 3,4-DNP and 3,5-DNP shown in Figure 15, and these salts were extremely insensitive in Table 7. Comparing with 3,4-DNP, **36** and **38** owned much lower decomposition temperatures, similar to that of **37**, **39** and 3,5-DNP. Zhang et al. [91] developed the ionic salts of LLM-116 with several nitrogen-rich cations as shown in Figure 16. These compounds showed extraordinary insensitivity to impact (>60 J), as the detonation properties of **40i** and **41k** were comparable to those of TATB (31.15 GPa, 8.11 km·s^−1^) (Table 7).

**Figure 17:**
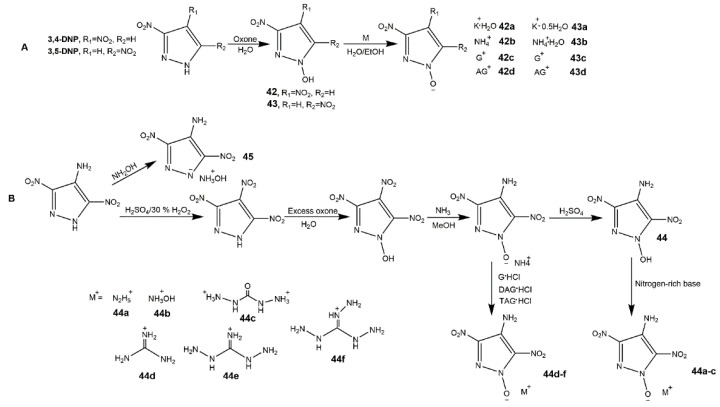
Synthesis of ionic salts of dinitropyrazoles.

*N*-oxidation of nitrogen-rich heterocycles including transformation of amino group to nitroso, azoxy, or nitro groups is another approach to designing HEDMs, which opens new avenues for the development of HEDMs [92,93]. The efforts to developing *N*-oxidation of dinitropyrazoles have been made recently. Bölter et al. [94] introduced -OH on *N* atom of 3,4-DNP and 3,5-DNP, and obtained several salts (Figure 17, Scheme A). From Table 8, these compounds were less sensitive than RDX, and did not exhibited excellent detonation properties. Yin et al. [95] synthesized a family of 4-amino-3,5-dinitro-1*H*-pyrazol-1-ol (**44**) and its ionic derivatives (**44a**–**f**) (Figure 17, Scheme B). Except **44**·H_2_O, all the compounds (**44a**–**f**, and **45**) with thermal decomposition temperatures (169–216 °C) shown good balance between detonation properties and insensitive properties as shown in Table 8. Zhang et al. [96] synthesized the 4-nitramino-3,5-dinitropyrazole by nitrating the -NH_2_ of LLM-116, and prepared several energetic salts which exhibited good insensitivity and moderate detonation properties.

**Figure 18:**
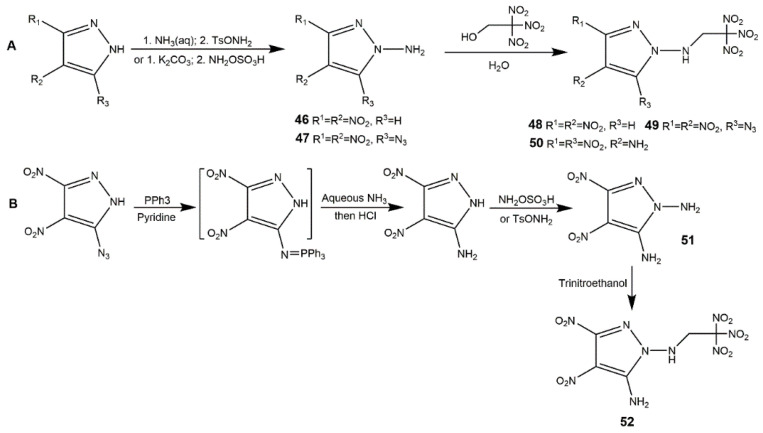
*N*-Trinitroethylamination of dinitropyrazole.

**Figure 19:**
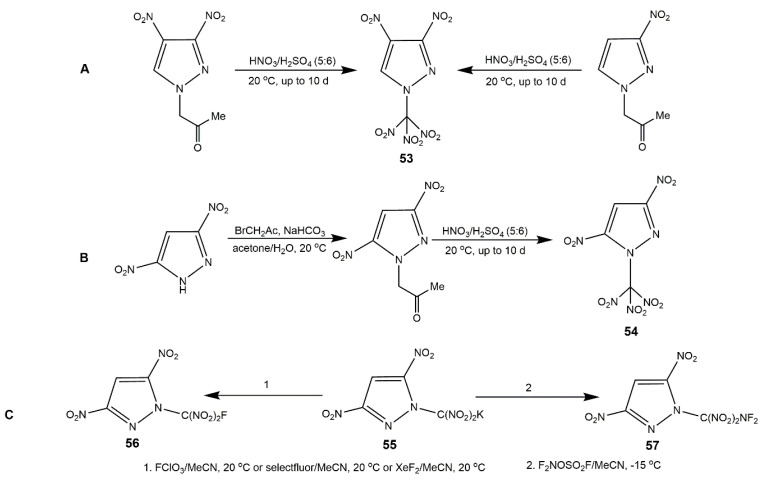
Synthesis of compounds **53**–**57**.

As mentioned above, polynitromethyl are considered to be more favorable groups to give remarkable improvements in densities and detonation properties of energetic materials. Especially the *N*-trinitroethylamination of nitropyrazole is more available since it is stable to be handled safely. The *N*-trinitroethylamination of dinitropyrazole was firstly proposed by Shreeve team [57]. They obtained several *N*-amino-dinitropyrazoles firstly, then these compounds underwent Mannich reactions with trinitroethanol to acquire the corresponding derivatives (**46**–**50**) (Figure 18, Scheme A). It was noteworthy that 1-amino-3,5-dinitropyrazole and 1-amino-3,4-dinitro-5-cyanopyrazole failed to get the corresponding compounds due to the electron-withdrawing effect of substituent groups bonded to dinitropyrazole ring. In addition, they employed an alternative synthetic method to obtain 1,5-diamino-3,4-dinitropyrazole (**51**) (Figure 18, Scheme B) because attempted amination of this compound using TsONH_2_ acid or NH_2_OSO_3_H failed. From Table 9, although the azido-functionalized dinitropyrazole (**47**) decomposed at 121 °C, compound **46** and **51** had high decomposition temperatures, and **47** and **50**–**52** owned higher density than RDX. These indicated the introduction of an -NH_2_ could enhance density. In addition, *N*-trinitroethylamination of dinitropyrazole (**48**–**50** and **52**) shown high HOF and good detonation properties. *N*-trinitromethyl moiety was introduced by Dalinger’s team [58], they synthesized 3,4-dinitro--1-(trinitromethyl)-pyrazoles (**53**) and 3,5-dinitro-1-(trinitromethyl)-pyrazoles (**54**) with excellent physical and computational properties as shown in Figure 19. They were a little less insensitive than the RDX and PETN, similar to *N*-trinitroethylamination dinitropyrazoles shown in Table 9. Fluorine and fluorinated functional groups are importantly promising substituents in the field of energetic materials [97]. C(NO_2_)_2_F and C(NO_2_)_2_NF_2_ moieties bring high energy, maintaining high density and good thermal property were incorporated into dinitropyrazole by fluorinated compound **55** (Figure 19, Scheme C). The two compounds had high density (≥1.92 g·cm^−3^), good oxygen balance (+2.55% for **57** and 0% for **56**), and high detonation pressure and velocity [98].

**Figure 20:**
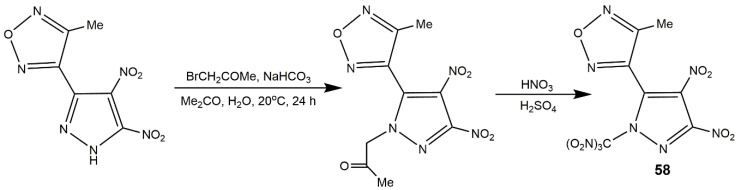
Synthesis of compound **58**.

**Figure 21:**
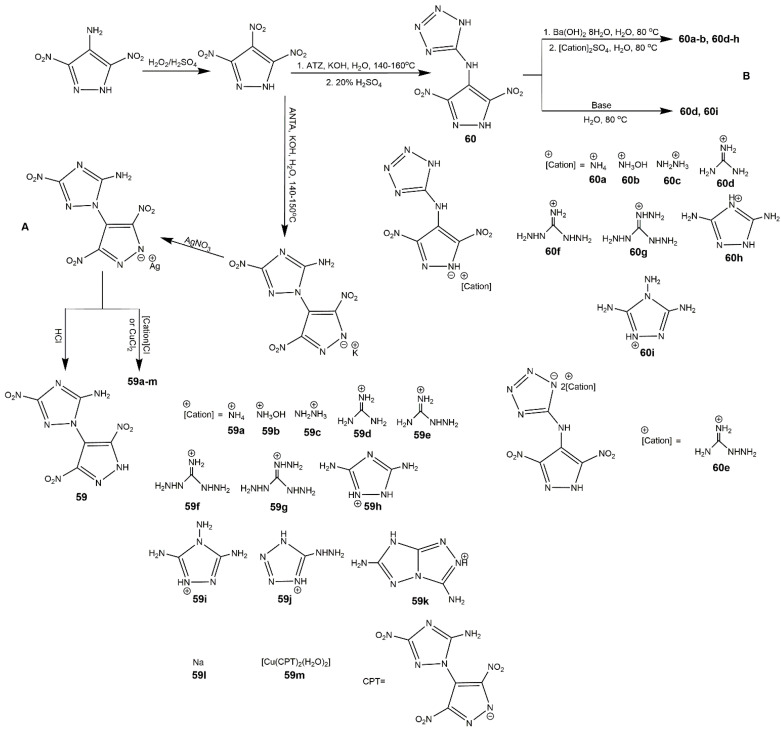
Synthesis of compounds **59**–**60i**.

Dinitropyrazoles bearing other heterocycles are also interesting and notable. To obtain the melt-castable explosives with good compatibility, improved oxygen balance and moderate detonation properties, compound **58** incorporating both *N*-trinitromethyl and *C*-methyl substituents in addition to nitro groups was synthesized by Sheremetev’s group [99] (Figure 20). This low melting temperature compound has been proved to own higher detonation pressure and velocity values than those of others melt-castable energetic heterocycles bearing methyl group, which provided feasible route to castable energetic materials. In addition, introduction of polynitrogen heterocycle and formation of energetic salts are main methods to improve the thermal stability of explosives [100]. In 2016, a heat-resistant energetic material, compound **59** bearing triazole ring, was synthesized using 5-amino-3-nitro-1*H*-1,2,4-triazole (ANTA) and 3,4,5-trinitrated-1*H*-pyrazole (TNP), and several salts based on it were developed by Zhou et al. [101] (Figure 21, Scheme A). As shown in Table 10, compound **59** had high decomposition temperature (270 °C) and high positive HOF (833 kJ·mol^−1^). All the salts showed good thermal stability, excellent insensitivity, and good detonation properties. In particular, the guanidinium salt **59d** exhibited the best thermal stability superior than that of most explosives. Considering thermal stability and energetic properties, compounds **59** and **59d** could be used as heat-resistant explosives and it was possible that these compounds can be applied as heat-resistant materials. Afterwards, their group reported a family of unsymmetrical *N*-bridged dinitropyrazoles synthesized by TNP and 5-amino-1*H*-tetrazole (ATZ) and its organic salts (Figure 21, Scheme B). Several compounds (**60**, **60b**, and **60c**) with high N contents exhibited superior detonation velocities but inferior detonation pressures compared to HMX and insensitivities to impact (IS > 40 J) and friction (FS > 360 N) comparable to those of TATB (Table 10), which could be promising insensitive HEDMs for practical application.

In summary, some dinitropyrazoles and derivatives exhibit low melting points and high decomposition temperatures as well as good detonation, which can make them competitive candidates for a castable explosive. To further improve the performance of dinitropyrazole-based energetic materials, a combination of several functional groups should be better, for example, the combination of nitramine and polynitrogen heterocyclic which can endow them with high thermal stability and good detonation performance.

### 2.3. Trinitropyrazole and Its Derivatives

TNP is the unique pyrazole compound by total carbon nitrification [102]. This compound owns good thermal stability (260–350 °C of *T*_d_) and chemical stability, and shows high detonation velocity (9.0 km·s^−1^) and detonation pressure (37.09 GPa). Wu et al. reviewed the synthesis of TNP in recent years in detail [102], including direct nitration methods, amino oxidation method, amino diazotization method, iodo nitrification method and microwave rearrangement method. The typical synthesis of TNP is the oxidation of LLM-116 rather than 5-amino-3,4-dinitropyrazole, and this is partly because the amino group in LLM-116 has higher electron cloud density and steric hindrance than amino group in 5-amino-3,4-dinitropyrazole, which can promote the intermolecular oxidation reaction and avoid the occurrence of intermolecular side reaction effectively, and partly because the “NO_2_-NH_2_-NO_2_” framework in LLM-116 makes it more stable and easier to synthesize. In addition, the nitrification of 3,5-DNP is another typical synthesis route of TNP. Traditional oxidation methods have the following defects: harsh reaction conditions, poor selectivity, by-products, high risk factor, expensive metal catalyst and toxic organic solvent. Although the synthesis of TNP with LLM-116 and 3,5-DNP as starting materials are mature, the synthesis of LLM-116 and 3,5-DNP are complicated. It is necessary to explore novel synthesis method. Zhao et al. [44] used LLM-116 as starting material, water as solvent, and KHSO_5_ as oxidant to synthesize TNP. Ravi et al. [103] put forward the nitration system of metal nitrate and studied the process of nitration to TNP. These two methods are promising to prepare TNP.

**Figure 22:**
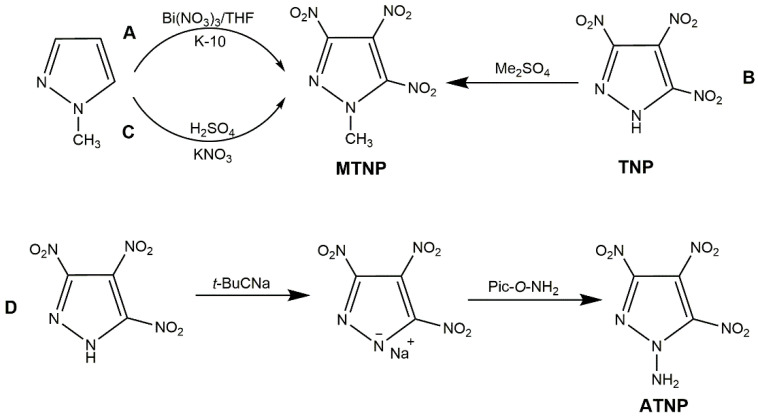
Synthesis of MTNP and ATNP.

Moreover, 1-methyl-3,4,5-trinitropyrazole (MTNP), a derivative of TNP, is an insensitive energetic material with 91.5 °C of melting point, 248–280 °C of decomposition temperature, 8.65 km·s^−1^ of detonation velocity, and 33.7 GPa of detonation pressure [104]. Ravi et al. [103] added K-10 and TNP to bismuth impregnated in THF to obtain MTNP (Figure 22, Scheme A). There were also many routes to synthesize MTNP. Dalinger et al. [105,106] dissolved TNP in NaHCO_3_ aqueous solution with Me_2_SO_4_ as methylation reagent to acquire MTNP (Figure 22, Scheme B). Guo et al. [107] synthesized MTNP from 1-methyl-pyrazole by one-step method with nitric acid and fuming sulfuric acid (Figure 22, Scheme C). Among these methods, selection of highly efficient catalytic synthesis process and low toxicity methylation reagent are the trend in MTNP synthesis. In addition, 1-amino-3,4,5-trinitropyrazole (ATNP) is also a derivative of TNP with excellent detonation properties (*D* = 9.17 km·s^−1^ and *P* = 40.9 GPa) and thermal stability [108]. This was reported by Herve et al. [93], and the synthesis route is shown in Scheme D of Figure 22 (Pic-*O*-NH_2_ = 2,4,6-trinitrophenyl-*O*-hydroxylamine) with a yield of 26%.

**Figure 23:**
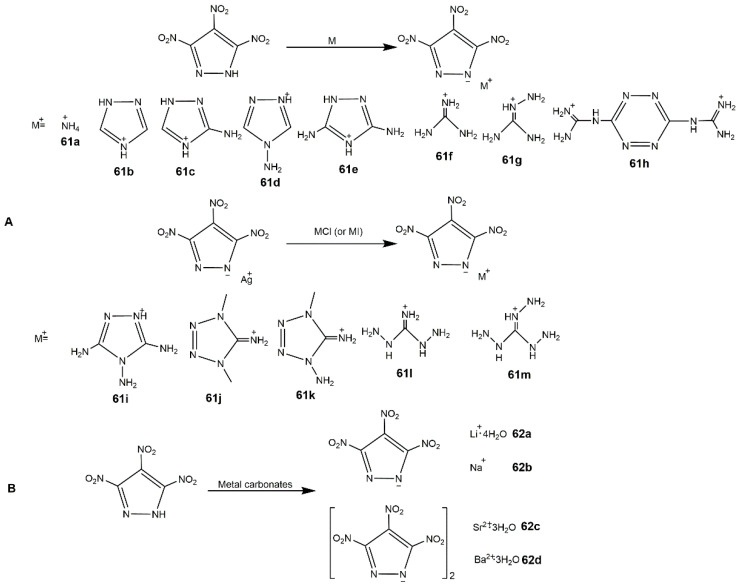
Synthesis of different salts of TNP. (**A**), the polynitrogen salts; (**B**), the alkali and earth alkali salts.

**Figure 24:**
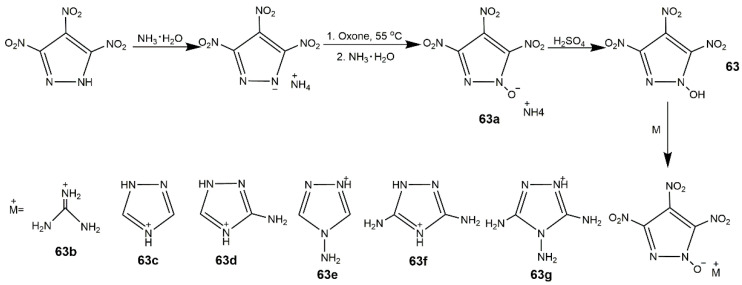
Synthesis of compound **63** and its salts.

The N-H bond in TNP is easy to neutralize with alkali or react with metal salts forming energetic salts due to the stereoscopic structure and spatial effect of pryazole ring. These energetic salts further broaden the application of TNP. Zhang et al. [109] prepared a series of energetic salts of TNP based on nitrogen-rich cations (**61a**–**m**) (Figure 23, Scheme A), all the salts showed poorer densities and detonation properties than TNP (Table 11), but they owned good thermal stability and excellent insensitivity. Drukenmuller et al. [110] reported the synthesis of alkali and earth alkali trinitropyrazolate (**62a**–**d**) (Figure 23, Scheme B), compound **62d** exhibited predominantly decomposition temperatures (Table 11). They also prepared pyrotechnic formulations using **62c** and **62d**, which showed good color properties and low sensitivity as well as high *T*_d_. In addition, Shreeve’s group [111] synthesized 3,4,5-trinitropyrazole-1-ol (**63**) and its nitrogen-rich salts (**63a**–**g**) (Figure 24) the corresponding properties are shown in Table 11. Compound **63** with its high oxygen content (51.13%) could be the green replacement of the currently used oxidizer (NH_4_ClO_4_), while the high IS (1 J) restricted its application. Compound **63a**–**g** with acceptable impact sensitivities and detonation performance could be useful energetic materials.

Polynitrogen heterocycle linking to TNP is a promising method to reach a balance between the energetic and physical properties of TNP, while there are a few references about it. Shreeve et al. [112] reported the synthesis of asymmetric *N*,*N*′-ethylene bridged 5-aminotetrazole and TNP moieties. They prepared 1-(2-(3,4,5-trinitro-1*H*-pyrazol-1-yl)ethyl)-1*H*-tetrazol-5-amine and 1-(3-(3,4,5-Trinitro-1*H*-pyrazol-1-yl)propyl)-1*H*-tetrazol-5-amine, and the two compounds were excellent insensitive and moderate powerful. In addition, they synthesized 5-((3,4,5-trinitro-1*H*-pyrazol-1-yl)methyl)-1*H*-tetrazole by *N*-methylene-*C* bridging TNP and tetrazole, which showed outstanding detonation properties and moderate insensitivity [113].

## 3. Nitrated-Bispyrazoles Based Compounds

Nitropyrazoles can be connected with nitrogen-rich heterocycles to obtain amazing energetic materials. In the previous section, nitropyrazoles bearing some polynitrogen heterocycles have been shown. Generally, these compounds exhibit some special properties, such as high detonation properties, good thermal stability, excellent safety, high density, and heat of formation, etc. Nitrated bispyrazoles also have attracted more and more attention, we will review the nitrated bispyrazole-based energetic materials in this section.

### 3.1. Directly Bridged Bis(Nitropyrazole)s

**Figure 25:**
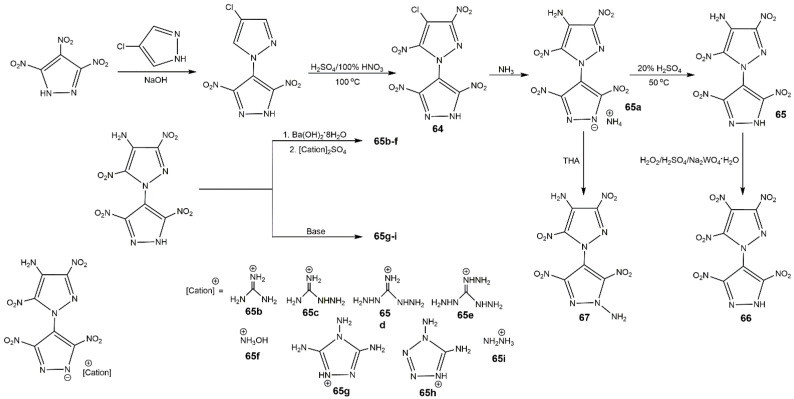
Synthesis of compounds **64**–**67**.

**Figure 26:**
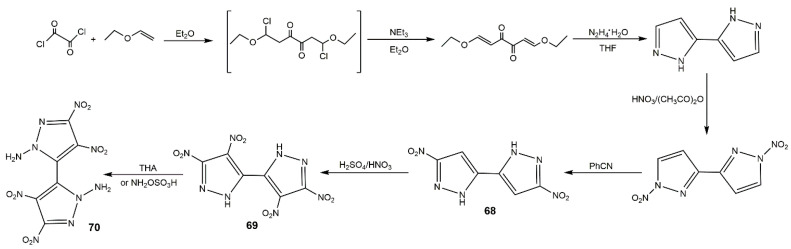
Synthesis of compounds **68**–**70**.

**Figure 27:**
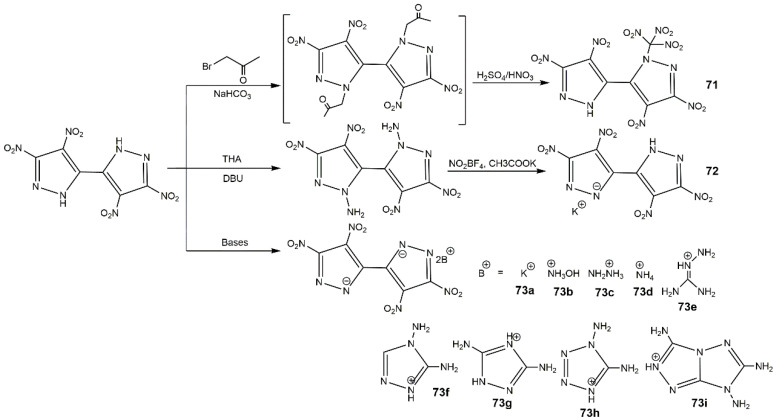
Synthesis of compounds **71–73i**.

**Figure 28:**
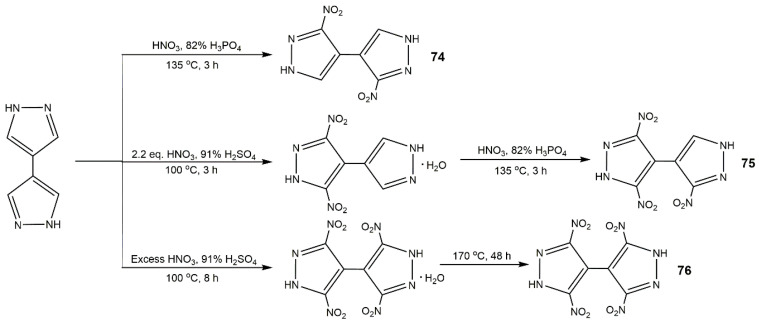
Synthesis of compounds **74**–**76**.

In 2014, Li et al. [27] synthesized several polynitro-substituted 1,4′-bridged-bispyrazoles energetic salts (**64**–**67**) as shown in Figure 25. They found that these compounds showed remarkable and unprecedented comprehensive properties (Table 12), and most of them with low toxicity were not hygroscopic. These compounds exhibited excellent impact sensitivities close to TATB, and the melting points and thermal decomposition temperatures were high, which could be applied as heat-resistant explosive. Compound **64** showed high *T*_d_ approximating that of hexanitrostilbene (HNS, 316 °C). The energetic properties of compounds **64**, **65**, **65a**, **66**, and **67** were comparable with or superior to RDX, especially compound **66**. In 2017, Tang et al. [114] prepared 4,4′,5,5′-tetranitro-2*H*,2′*H*-3,3′-bipyrazole (**69**) and its di-*N*-amino product (**70**), and the detailed route is described in Figure 26. Compound **70** showed good thermal stability and insensitivities as well as high detonation properties (Table 12). In addition, they synthesized 4,4′-dinitro-5,5′-diamino-2*H*,2′*H*-(3,3′-bipyrazole) (consisting of two 3-amino-4-nitropyrazole rings), this compound also show outstanding balance between thermal stability and safety (Table 12) [115]. Afterwards his team reported a variety of energetic materials based on compound **69** shown in Figure 27. Compounds **71**, **73b**, and **73h** had high densities and good detonation velocities (Table 12), which were superior to RDX suggesting their use in secondary explosives. The dipotassium salt **73b** had a high density of 2.029 g·cm^−3^ and excellent thermal stability of 323 °C, and could be applied as primary explosives [116]. However, the poor impact sensitivity might restrict their further application. In 2019, Domasevitch and co-authors [117] found an efficient approach towards facile accumulation of nitro functionalities at the pyrazole platform. Compounds **74**, **75**, and **76** were synthesized according to Figure 28. From Table 12, the three compounds owned high decomposition temperatures above 290 °C, especially for **75** and **76**. The introduction of three and four -NO_2_ into the 4,4-bipyrazole scaffold could produce insensitive and thermally stable energetic materials with ideal densities and good detonation properties.

### 3.2. Alkyl-Bridged Bis(Nitropyrazoles)

**Figure 29:**
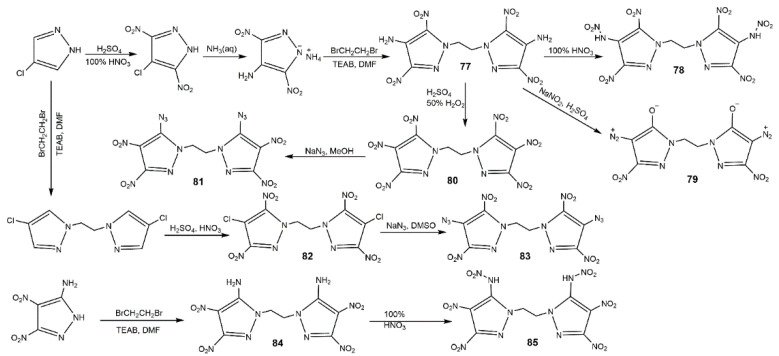
Synthesis of bis(nitropyrazoles) linked by *N*,*N′*-ethylene-bridge **77**–**85**.

**Figure 30:**
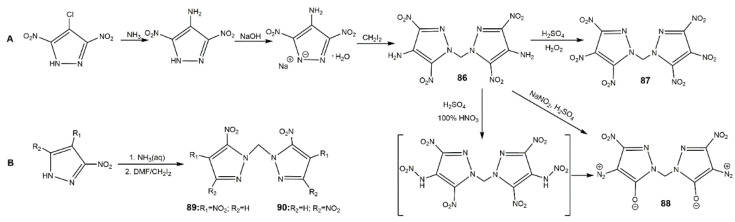
Synthesis of bis(nitropyrazoles) linked by *N*,*N′*-methylene-bridge **86**–**90**.

**Figure 31:**
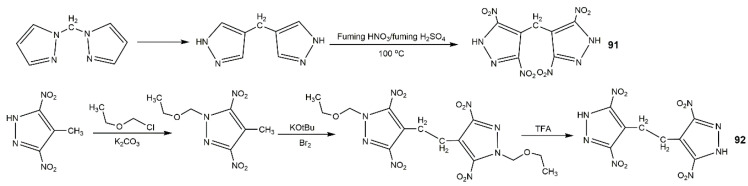
Synthesis of compounds **91** and **92**.

Alkyl is also a good linkage to construct nitrogen-rich moieties, and many *N*,*N′*-alkyl-bridged energetic materials have been developed [112,118,119,120,121]. Yin et al. [122] developed a novel class of *N*,*N*′-ethylene-bridged bis(nitropyrazoles) with the synthetic route shown in Figure 29. Compounds **77**–**85** displayed various properties (Table 13) owing to the diversified functionalizations. Diaminobis(pyrazoles) showed good thermal stability, highly insensitivity, and favorable energetic performance; for example, the thermal decomposition temperature (311 °C) and detonation properties (27.9 GPa and 8.19 km·s^−1^) of **77** were higher than those of TNT, and were comparable to those of TATB. By contrast, *N*,*N*′-ethylene bridged dinitraminobis(pyrazoles) and diazidobis(pyrazoles) owned better detonation performances, while having higher impact and friction sensitivity. Compound **80** was the most promising energetic material with high density, favorable thermal stability, and good detonation properties, which were comparable to RDX. In addition, the relatively low impact and friction sensitivities of **80** showed good integrated properties, highlighting its potential application as a replacement of RDX. In 2016, Fischer et al. [123] synthesized three different bisnitropyrazole-based energetic materials by *N*,*N′*-methylene bridge (**86**–**88**), the detailed synthetic route is displayed in Scheme A of Figure 30. These energetic compounds could be used for different applications according to their properties (Table 13), compound **86** was a secondary explosive with a high *T*_d_ (310 °C), enhanced detonation parameters by contrast with HNS, and high sensitivity to external stimuli. Compound **87** exhibited excellent detonation velocity (approximately to CL-20). The higher performance and better thermal stability of **88** was relative to DDNP making it a potential candidate as a green primary explosive. In addition, the synthetic routes are economical. Afterwards, their group used a similar route to prepare bis(3,4-dinitro-1*H*-pyrazol-1-yl)methane (**89**) and bis(3,5-dinitro-1*H*-pyrazol-1-yl)methane (**90**) with high decomposition temperature and low sensitivities having capability as future energetic materials (Table 13) [94]. Gozin et al. [124] explored the possible influence factor of the thermostable property of explosives, and under the guidelines they proposed, they synthesized the compounds **91** and **92** with excellent thermal stability and moderate sensitivities shown in Figure 31 and Table 13.

### 3.3. Ring-Bridged Bis(Nitropyrazoles)

**Figure 32:**
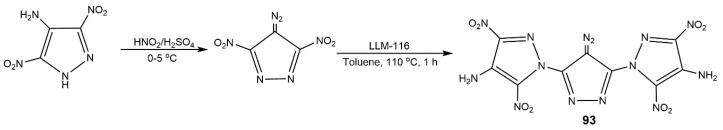
Synthesis of compound **93**.

**Figure 33:**
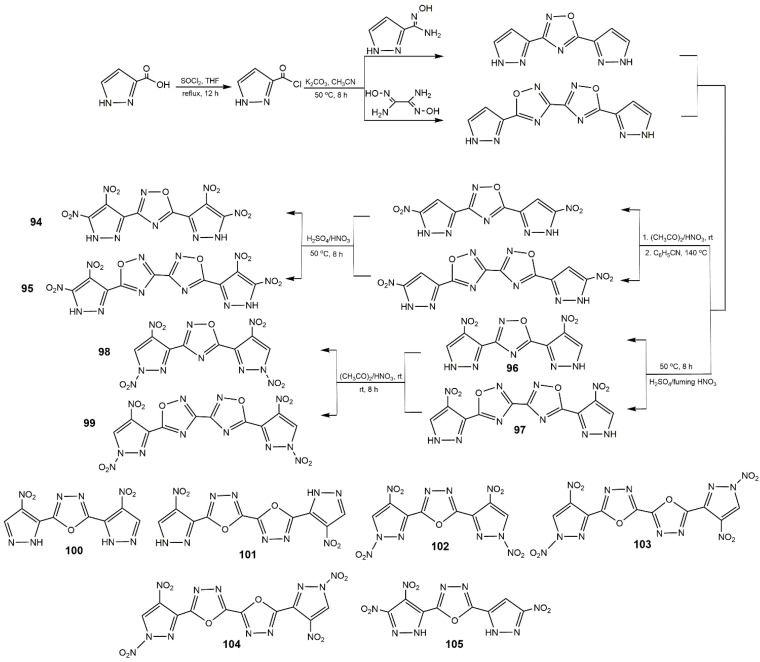
Synthesis of compounds **94**–**99**, and chemical structures of compounds **100**–**105**.

**Figure 34:**
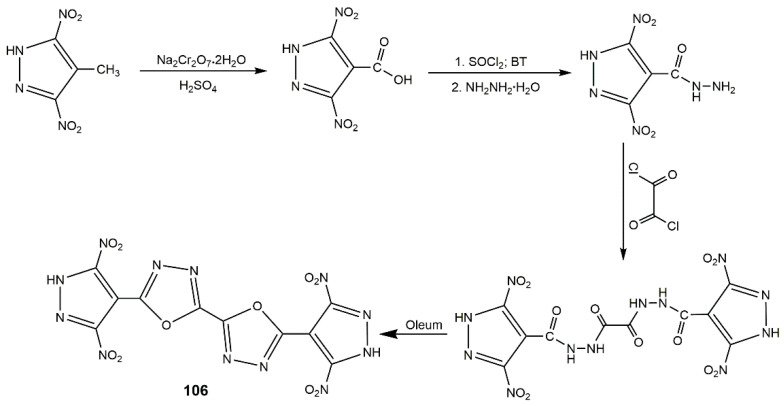
Synthesis of compound **106**.

Ring-bridge is an important connector linking bis(nitropyrazoles) to obtain high performance energetic materials. Pagoria et al. [125] reported the trimerization of LLM-116. 4-Diazo-3,5-bis(4-amino-3,5-dinitropyrazol-1-yl) pyrazole (**93**) containing a stable diazo group was synthesized, and the detailed route is shown in Figure 32. Compound **93** was more thermally stable (278 °C of *T*_d_) than LLM-116, attributing to the considerable hydrogen bonding between -NH_2_ and -NO_2_, and the short contact between the =N_2_ and -NO_2_ through the intermolecular interactions. Moreover, it was insensitive to impact, friction, and spark. Yan et al. [126] designed mono and bi(1,2,4-oxadiazole) rings to bridge polynitropyrazoles (Figure 33). Among compounds **94**–**99**, **98**, and **99** owned the highest detonation velocity of 8.90 and 8.87 km·s^−1^, detonation pressure of 35.1 and 34.5 GPa, respectively. **94** and **95** processed good stability (272–274 °C) and good insensitivity (IS > 30 J and FS > 360 N) as well as high detonation properties (8.69–8.74 km·s^−1^ of *D* and 33.4–34.0 GPa of *P*). **96** and **97** had the high thermal stability over 310 °C and good sensitivity (IS > 40 J, FS > 360 N). Comparing with the conventional heat resistant explosive HNS, **96** and **97** owned better detonation properties (7.99–8.03 km·s^−1^ of *D*, 25.2–26.4 GPa of *P*). Also, their team used the similar routes to synthesize the bis(nitropyrazoles) with 1,3,4-oxadiazole (**100**–**105**) [127]. The properties of these compounds are showed in Table 14. Moreover, Li et al. [124] synthesized the compound **106** with the procedure shown in Figure 34. This compound exhibited an excellent decomposition temperature (341 °C), high calculated detonation velocity of 8.52 km·s^−1^, and detonation pressure of 30.6 GPa. It also showed impressive insensitivities (IS = 22 J, FS = 352, and ESD = 1.05 J). These showed building ring bridged bis(nitropyrazoles) can be an effective approach to enhance the properties of energetic materials.

**Figure 35:**
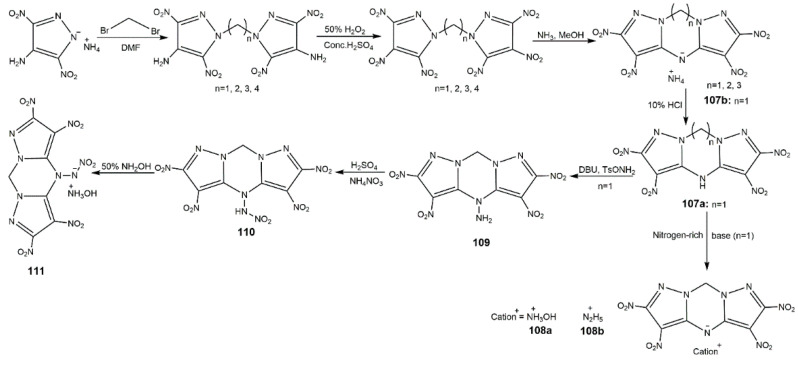
Synthesis of compounds **107a**–**111**.

**Figure 36:**
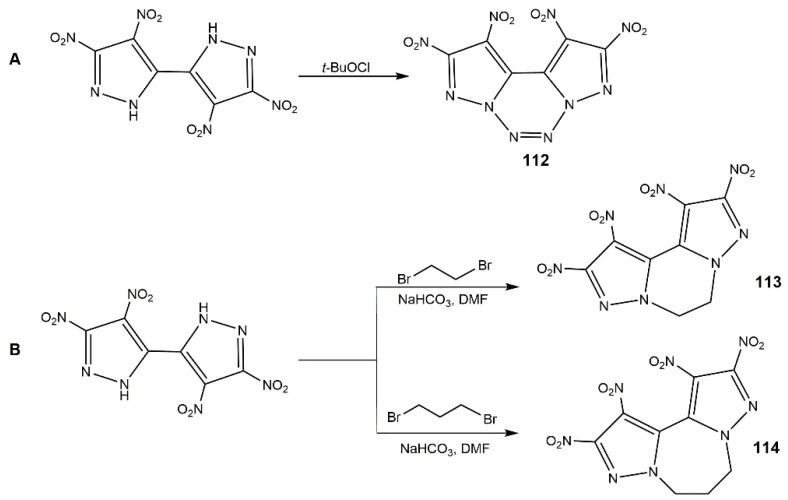
Synthesis of compounds **112**–**114**.

In addition, there are some other fused ring-bridged bis(nitropyrazoles). In 2017, Yin and co-authors [128] synthesized compound **109** and its derivatives according to the procedure shown in Figure 35, and their physicochemical and energetic properties are shown in Table 15. Among these compounds, **107a** had a high density and decomposition temperature as well as the good safety parameters. The introduction of nitramino group gave **110** and **111** highest detonation velocities and pressures, while they also exhibited sensitive properties to mechanical stimuli. Considering the whole aspect, **108a** was featured with promising integrated energetic performance exceeding those of the benchmark explosive RDX. Shreeve’s group prepared (**112**) obtained from compound **69** by *N*-azo coupling reactions shown in Scheme A of Figure 36 [114]. Compound **112** had a high density of 1.955 g·cm^−3^ and a good thermal stability (233 °C). Its detonation properties (9.63 km·s^−1^ and 44.0 GPa) were comparable to CL-20, much better than those of RDX and HMX. In addition, the IS of 10 J and FS of 240 N showed it was more stable than CL-20. These indicated compound **112** was a superior energetic explosive. In 2018, her team developed an efficient synthetic method of ring closure of polynitropyrazoles with *N*,*N*′-ethylene/propylene bridges (Figure 36, Scheme B). Compounds **113** and **114** showed excellent thermal stability (261 °C for **113**, 280 °C for **114**), good detonation properties and moderate insensitivities, making them potential candidates as HEDMs. This ring closure strategy could provide new ideas of designing thermally stable explosives.

### 3.4. DCNP-Bridged Bis(Nitropyrazoles)

**Figure 37:**
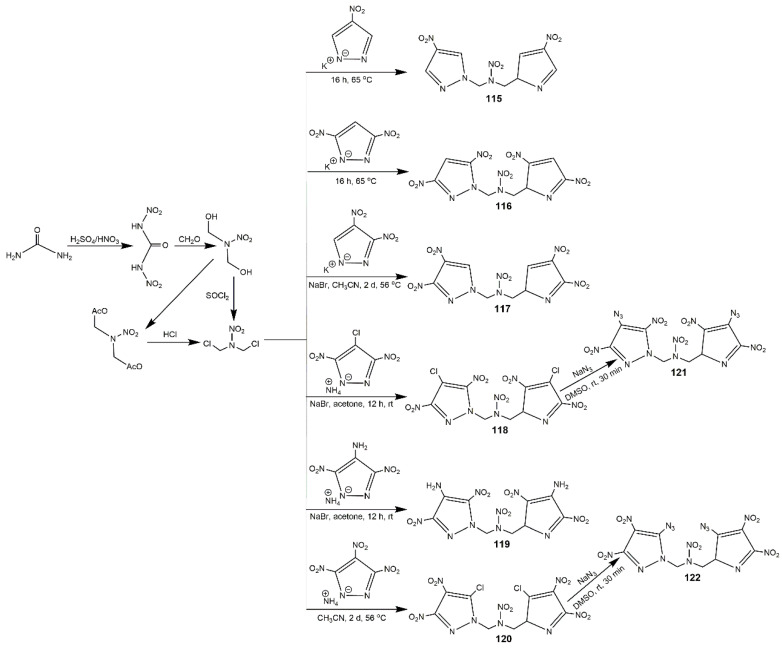
Synthesis of nitrated bispyrazoles **115**-**122** from 1,3-dichloro-2-nitro-2-azapropane.

It is known that 1,3-Dichloro-2-nitro-2-azapropane (DCNP) is an useful precursor connecting nitropyrazoles via nucleophile substitution [129]. In 2013, Zhang et al. [130] reported a family of functionalized dipyrazolyl *N*-nitromethanamines (compounds **115**–**122** in Figure 37) using DCNP as the bridge. These compounds exhibited densities between 1.69–1.90 g·cm^−3^ and thermal stabilities range from 166–354 °C. From Table 16, it was easy to see the introduction of the azidodinitropyrazolate group led to the most competitive detonation properties (35.1 GPa and 8.72 km·s^−1^ for **121**, 35.2 GPa and 8.72 km·s^−1^ for **122**). However, they showed high sensitivity (IS = 2 J). Compound **119** exhibited good physical and detonation properties, such as high thermal stability, density, HOF, detonation pressure and velocity, and great impact stability, which could be used a promising HEDM. Klapötke et al. [131] also reported these compounds. They applied a different synthesis method of DNCP by the nitration of hexamethylenetetramine, and the NaBr/acetone system was used to substitution reaction.

In general, the physical and energetic properties of bridged bis(nitropyrazole)s can be adjusted by the bridged groups. The design of novel bridged group would be a key factor to synthesize new HEDMs, and forming polycyclic derivatives even cage compounds could be more attractive. In addition, the salts of bridged bis(nitropyrazole)s should be explored in-depth.

## 4. Nitrated Pyrazolo[4,3-*c*]Pyrazoles and Their Derivatives

Application of molecular design and explosive performance prediction has explored many novel energetic materials based on pyrazolopyrazole ring system [2,132,133]. Heterocycles like pyrazolo-pyrazole always own high density and oxygen balance, good thermal stability, and enhanced energetic performance of an energetic material.

**Figure 38:**
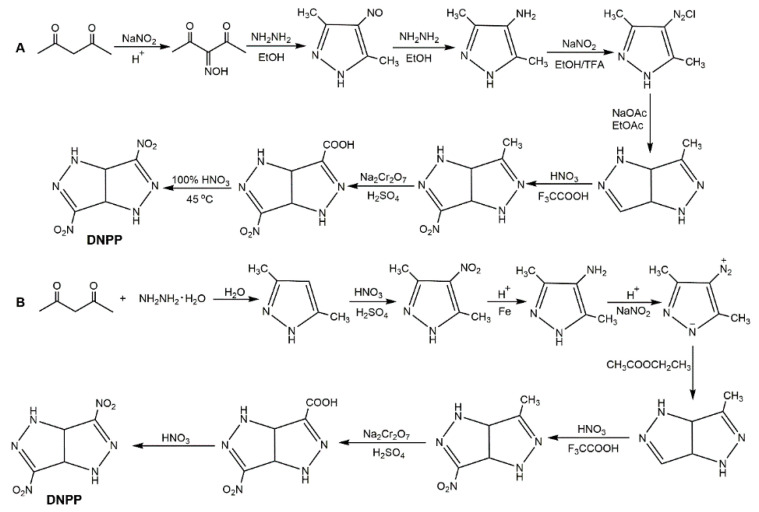
Synthesis of DNPP.

3,6-Dinitropyrazolo[4,3-*c*]pyrazole (DNPP) is a new type of energetic material with attractive properties (1.865 g·cm^−1^ of *ρ*, 42.42% of nitrogen content, 273 kJ·mol^−1^, 330.8 °C of *T*_d_ and 68 cm of *D*_50_). This compound synthesized from 3,5-dimethylpyrazole was firstly reported by Dalinger and co-workers [134]. Pagoria et al. [135] improved the synthetic route to DNPP as shown in Scheme A of Figure 38. In this procedure, 4-diazo-3,5-dimethylpyrazole salt is an important intermediate. Li et al. [136] improved the process of 4-diazo-3,5-dimethylpyrazole salt using freezing crystallization instead of extraction which avoided large use of organic solvents and improved its yield. This procedure has several advantages, such as ease of synthesis scale-up and better product yield. In addition, Luo et al. [137] proposed that DNPP could be obtained by dehydration condensation, primary nitration, reduction, diazotization, cyclization, secondary nitration, oxidation, and decarboxylation nitration with acetylacetone and hydrazine hydrate as raw materials (Figure 38, Scheme B).

**Figure 39:**
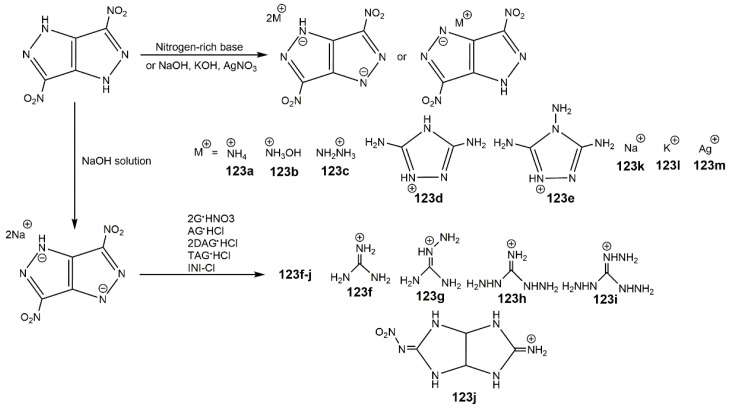
Synthesis of salts of DNPP.

Due to the active N-H bond in molecule of DNPP, it is easy to obtain its energetic salts. In 2014, Zhang et al. [138] reported a series of nitrogen-rich energetic salts based on the anion of DNPP (**123a**–**m**) shown in Figure 39. Salts **123a**–**e** could be obtained by reacting DNPP with ammonia, hydrazine, hydroxylamine, 3,5-diamino-1,2,4-triazole, and 3,4,5-triamino-1,2,4-triazole. Salts **123f**–**j** could be synthesized by reacting Na_2_DNPP with guanidine nitrate, aminoguanidine, diaminoguanidine, triaminoguanidinium, and 2-iminium-5-nitriminooctahydroimidazo [4,5-d]imidazole hydrochlorides. Salts **123k**–**m** were acquired by the reaction of DNPP with NaOH, KOH and AgNO_3_ respectively. Table 17 displays the properties of these energetic salts. It was notable that the ammonium salt (**123a**), hydroxylammonium salt (**123b**) and guanidinium salt (**123f**) exhibited outstanding decomposition temperatures of >300 °C. Furthermore, the sodium salt (**123k**) and potassium (**123l**) salt of DNPP were thermally stable up to 395 °C and 365 °C, respectively. In addition, most of the salts showed high calculated detonation properties, especially **123b** owned the highest detonation velocity and pressure. Considering the balance of safety and energetic properties as well as physical properties, **123b** could be a competitive candidate in insensitive HEDMs. Luo and co-authors synthesized the basic lead salt of DNPP (Pb-DNPP) and the 3,6-dihydrazine-1,2,4,5-tetrazine salt of DNPP (DHT-DNPP), and studied their thermal decomposition behaviors. Like **123k**–**m**, the introduction of heavy cations made the salts higher densities and *T*_d_. Combining other organic amines salts of DNPP [139], these salts showed good thermal stabilities.

**Figure 40:**
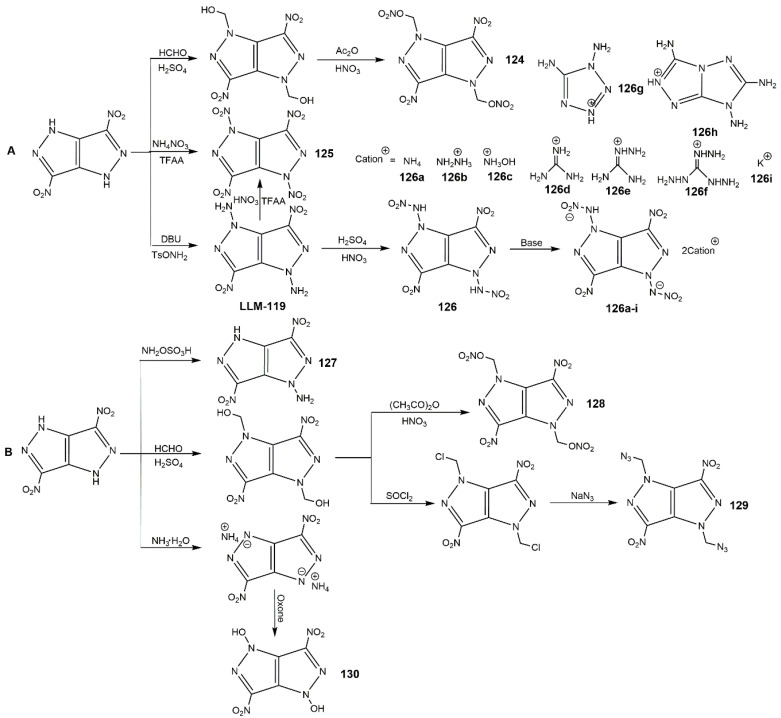
Synthesis of *N*-functional derivatives of DNPP.

In addition, 1,4-Diamino-3,6-dinitropyrazolo[4,3-*c*]pyrazole (LLM-119) is a derivative of DNPP with a predicted energy of 104% HMX and good insensitivity to friction and electric spark stimulation [2]. It is also a very important intermediate of synthesizing novel high-performance energetic materials. Li et al. [140] used NaOH and H_2_NOSO_3_H to realize the *N*-amination of DNPP, while the yield was low (10.4%). Yin reported a modified procedure using 1,8-diazabicycloundec-7-ene (DBU) and *O*-tosylhydroxylamine (TsONH_2_) as organic solvents with a good yield [141]. He also developed a series of DNPP derivatives based on *N*-functionalization strategy including several ionic salts of DNPP, the synthesis route is displayed in Figure 40 (Scheme A). As shown in Table 18, compounds **125**, **126** and **126c** exhibited high densities and excellent detonation velocities and pressures, which were superior to the current secondary explosive benchmark HMX. These compounds except **126d** and **126e** were sensitive to stimulation, especially for **126i** also showed excellent density and good thermal stability. These could make compound the potassium salt as a green primary explosive. Compounds **126a**, **126b**, **126c** and **126g** showed good possibilities for application in bipropellants owing to the high values of (N + O) content and specific impulse. Li and co-author [142] synthesized another four kinds of neutral explosives based on *N*-functionalization of DNPP shown in Scheme B of Figure 40. Comparing with LLM-119, compound **127** showed slightly lower energetic and physical properties due to the only one -NH_2_. Compounds **130** owned the relatively high density, good thermal stability, outstanding detonation properties, and reasonable sensitivities, which could be a useful energetic material. Li et al. [143,144] also synthesized several salts of *N*-nitramino DNPP, which exhibited good energetic properties.

**Figure 41:**
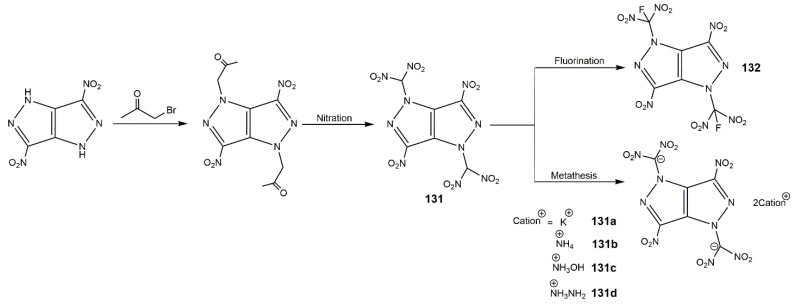
Synthesis of derivatives of DNPP containing dinitromethyl and fluorodinitromethyl group.

**Figure 42:**
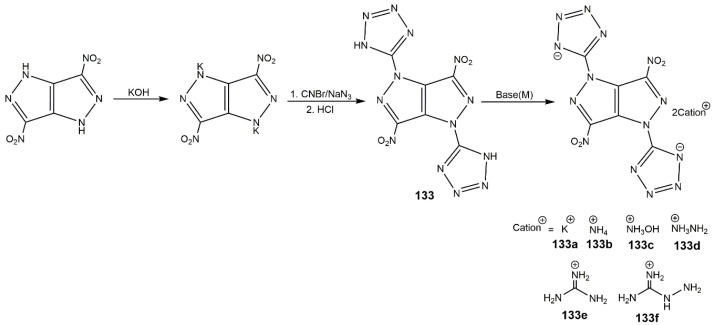
Synthesis of derivatives of DNPPP containing tetrazole groups.

In addition, Zhang et al. [145] introduced the dinitromethyl group and fluorodinitromethyl group into DNPP molecule and synthesized five fused-ring energetic derivatives (**131**–**132**) shown in Figure 41. Among these compounds, the dipotassium salt (**131a**) was formed as an interesting three-dimensional metal-organic framework (MOF) and exhibited outstanding detonation performances (9.02 km·s^−1^ of *D* and 33.6 GPa of *P*), which were comparable to that of Pd(N_3_)_2_. The compound **132** had a high density of 1.939 g·cm^−3^, high decomposition temperature of 213 °C and desired mechanical sensitivities (IS: 12 J; FS: 240 N), which could be a competitive candidate of RDX. These energetic compounds containing dinitromethyl or fluorodinitromethyl group enrich the energetic compound library of pyrazolo[4,3-*c*]pyrazoles. Furthermore, their group incorporated two tetrazole groups into DNPP molecule, and synthesized 3,6-dinitro-1,4-di(1*H*-tetrazol-5-yl)-pyrazolo[4,3-*c*]pyrazole (**133**) and its ionic derivatives (**133a**–**f**) shown in Figure 42 [146]. The physicochemical and energetic properties of these compounds are shown in Table 19. These compounds were thermally stable and insensitive to mechanical stimulation. The potassium salt (**133a**) possessed a high thermal decomposition temperature (329 °C of *T*_d_) and low sensitivities (IS: 25 J; FS: 252 N). In contrast with other derivatives from DNPP, compound **133f** owned the best mechanical sensitivities (IS: >60 J; FS: >360 N). Compounds **133**, **133a**, and **133d** possessed good comprehensive properties, including remarkable thermal decomposition temperatures, excellent insensitivity, and favorable detonation performance.

Nitrated pyrazolo[4,3-*c*]pyrazoles own acceptable performances both the energetic and physical properties, further functionalization of these compounds could be interesting. However, the synthesis of DNPP are still multistep reactions with unsatisfactory yield. The more efficient and facile synthesis technology should be investigated.

## 5. Conclusions

In recent years, a lot of scholars over the world have paid much attention to the development of nitrogen-rich heterocyclic energetic materials, due to their high positive heat of formation, low sensitivity, tailored thermal stability, and attractive detonation performance. According to the reference [37], a new energetic compound should be environmentally friendly, easy and economical to synthesize, thermal stable (*T*_d_ > 200 °C), insensitive to mechanical stimulation (IS > 7 J; FS > 120 N), good detonation properties (*D* > 8.5 km·s^−1^), and not insoluble in water. For the nitropyrazoles-based energetic materials, most of them can meet these requirements. Some nitropyrazole-based compounds show good performance as castable explosives, such as compounds **1**, **2**, **3**, **5**, 3,4-DNP, **46**, **48**, **53**, **54**, and MTNP, which are competitive candidates of TNT. Some exhibited excellent thermal stability such as compounds **17**, **28**, **37**, **64**, **75**, **86**, **90**, **92**, **101**, **120**, DNPP, etc. Further, many showed a balance between good safety and high detonation performance. The introduction of high-nitrogen groups (including fused-ring, polynitramino group, polynitromethyl group, etc) to nitropyrazoles can be useful approach for the further development of new-generation HEDMs. In addition, the concept of forming ionic salts, bridged structures and pyrazolo-pyrazoles provides novel insights to synthesize high performance energetic materials. It is better to synthesize new energetic compounds under the direction of theoretical calculation, so it is important to understand the relationship between structures and properties for the design and synthesis of new nitropyrazoles-based energetic materials.

Furthermore, there are some areas requiring improvement for the further synthesis of novel nitropyrazoles-based EMs. First, traditional nitration is generally used in the synthesis of nitropyrazoles-based EMs, which does not meet the requirements of modern green chemistry. It is vital to find out the suitable green nitrating agents and catalysts in the future synthesis process. Second, many syntheses of nitropyrazoles-based EMs entail several steps, leading to a low yield and high cost. Therefore, it is necessary to search for an efficient route when preparing new HEDMs.

## Figures and Tables

**Table 1:** Properties of 3-NP, 4-NP, 3-MNP and 4-MNP.

Explosive	*ρ*/g·cm^−3^	*D*/km·s^−1^	*P*/GPa	*T*_m_/°C	OB/%	N/%	Ref.
3-NP	1.57	7.02	20.08	174–175	−77.88	37.17	[55]
4-NP	1.52	6.86	18.81	163–165	−77.88	37.17	[55]
3-MNP	1.47	6.62	17.11	80–83	−107.09	26.77	[3]
4-MNP	1.40	6.42	15.52	82	−107.09	26.77	[3]

**Table 2:** Properties of the derivatives of compounds **1**–**7**.

Entry	*ρ*/g·cm^−3^	*D*/km·s^−1^	*P*/GPa	*T*_m_/°C	*T*_d_/°C	OB/%	N/%	HOF/kJ ·mol^−1^	IS/J	FS/N	Ref.
**1**	1.74	8.39	30.8	109.0	112	+2.75	33.68	140.9	15.0	360	[57]
**2**	1.80	8.65	33.9	39.0	145	−6.10	32.07	181.0	7.5	120	[58]
**3**	1.85	8.93	35.9	59.9	113	+18.3	32.07	311.4	2.5	36	[56]
**4**	1.80	8.60	32.2	147.2	154	+18.3	32.07	208.1	3.0	80	[56]
**5**	1.90	9.12	37.2	74	124	+24.8	34.79	320.2	5.0	80	[59]
**6**	1.84	8.79	33.8	-	128	−9.64	39.35	194.8	7.0	192	[61]
**7**	1.76	8.53	30.8	-	117	−25.79	32.26	205.6	17.0	114	[62]
**7a**	2.01	8.13	29.5	-	171	−21.94	27.44	−55.7	4.0	36	[62]
**7b**	1.87	8.26	28.6	-	203	−23.42	29.29	28.2	9.0	120	[62]
**7c**	1.78	8.60	33.0	-	141	−34.16	35.90	85.4	>20.0	192	[62]
**7d**	1.80	8.70	34.1	-	166	−24.20	33.60	135.5	>20.0	162	[62]
**7e**	1.72	8.26	28.7	-	161	−46.37	40.58	43.8	>20.0	240	[62]
**7f**	1.71	8.48	30.1	-	140	−46.74	43.29	231.4	>20.0	252	[62]
TNT	1.65	6.88	19.5	80. 5	295	−73.8	18.49	−67.0	15.0	358	[62]
RDX	1.80	8.75	34.9	204.1	210	−21.6	37.82	70.0	7.0	120	[62]

**Table 3:** Energetic characteristics of compounds **8**–**15**. The data of compounds **8**–**11** are from reference [66], the data of compounds **12**–**15** are from reference [67].

Entry	*ρ*/g·cm^−3^	*D*/km·s^−1^	*P*/GPa	*T*_d_/°C	N/%	HOF/kJ· mol^−1^	IS/J	FS/N
**8**	1.79	8.86	34	127	42.43	602	-	-
**9**	1.81	8.47	31	111	41.59	386	-	-
**10**	1.91	8.99	36	138	41.67	589	-	-
**11**	1.76	8.78	32	132	42.43	629	-	-
**12**	1.76	8.26	25.9	272	59.70	408	30	360
**12a**	1.78	8.82	29.9	187	56.89	445	32	360
**12b**	1.75	8.80	28.9	229	63.36	547	35	>360
**12c**	1.70	8.29	24.9	251	61.39	399	40	>360
**12d**	1.69	8.24	23.9	224	63.84	486	40	>360
**12e**	1.72	8.14	23.6	287	63.21	338	40	>360
**13**	1.72	8.00	22.8	200	52.96	127.6	>40	>360
**13a**	1.68	8.00	23.8	196	45.15	−483.3	32	360
**14**	1.75	8.81	32.6	148	47.94	462.8	30	360
**15**	1.78	7.79	21.8	406	53.52	127.1	>40	>360

**Table 4:** Energetic characteristics of compounds **16a**–**21d**. The data of compounds **16a**–**d** are from reference [69], the data of compounds **17**–**19i** are from reference [70], the data of compounds **20a**·H_2_O –**21d**·H_2_O are from reference [71].

Entry	*ρ*/g·cm^−3^	*D*/km·s^−1^	*P*/GPa	*T*_d_/°C	HOF/kJ· mol^−1^	IS/J	FS/N
**16a**	1.90	9.39	40.0	155.0	74	6	120
**16b**	1.87	9.58	38.5	194.0	266	12	160
**16c**	1.80	8.84	32.6	192.0	−20	15	240
**16d**	2.12	7.64	26.4	232.0	246	2	120
**17**	1.77	8.24	23.1	353.6	555.0	>40	>360
**18**	1.87	8.75	33.0	238.2	737.6	30	360
**19**	1.92	9.01	35.9	134.4	791.8	20	270
**19a**	1.76	8.68	30.0	186.6	711.4	>40	>360
**19b**	1.79	9.08	33.6	171.3	842.0	>40	>360
**19c**	1.73	8.76	30.2	186.6	1062.4	>40	>360
**19d**	1.71	8.19	25.1	195.4	677.9	>40	>360
**19e**	1.71	8.50	27.3	191.3	1068.7	>40	>360
**19f**	1.75	8.71	29.7	208.2	1014.6	22.4	>360
**19g**	1.72	8.12	25.7	168.5	1300.5	>40	>360
**19h**	1.74	8.16	26.0	189.7	1270.5	>40	>360
**19i**	1.72	8.14	25.9	175.9	1511.2	>40	>360
**20a**	1.82	8.39	28.2	180.0	60.0	>40	360
**20b**	1.83	8.10	28.0	279.0	105.0	40	240
**21**	1.89	8.71	31.9	248.0	314.6	>40	>360
**21a**·H_2_O	1.95	8.29	29.1	341.0	260.9	>40	>360
**21b**·H_2_O	1.81	8.98	32.1	218.0	386.2	>40	>360
**21c**·H_2_O	1.80	9.06	31.7	190.0	557.5	>40	>360
**21d**·H_2_O	1.60	8.22	24.6	223.0	690.0	>40	>360

**Table 5:** A brief comparison of four routes by Stefan.

Via 4-NP (Method A)	Via 3,5-Dimethylpyrazole (Method B)	Via 3,5-DNP (Method C)	Via 4-Chloropyrazole (Method D)
Four steps	Six steps	Five steps	Two steps
Moderate amount of waste	High amount of waste	Moderate amount of waste	Small amount of waste
No unfavorable solvents required	No unfavorable solvents required	DMSO used in the last step	No unfavorable solvents required
Moderate overall yield, 40%	Moderate overall yield, 37%	Low overall yield, 21%	Moderate overall yield, 61%
Average yield/step: 80%	Average yield/step: 85%	Average yield/step: 73%	Average yield/step: 78%

**Table 6:** Physical and detonation properties of compounds **22**–**35**. The data of compounds **22**–**35** are from reference [90].

Entry	*ρ*/g·cm^−3^	*D*/km·s^−1^	*P*/GPa	*T*_d_/°C	HOF/kJ· mol^−1^	IS/J
**22**	1.74	8.22	30.1	178	137.0	17
**23**	1.69	8.25	28.7	176	104.6	35
**24**	1.72	8.31	30.2	176	220.7	18
**25**	1.63	8.14	26.3	275	64.8	>60
**26**	1.88	8.73	35.0	241	166.0	>40
**27**	1.66	7.82	23.4	245	133.5	>40
**28**	1.84	8.46	31.0	308	182.6	>40
**29**	1.78	8.39	31.4	233	236.6	10
**30**	1.74	8.41	31.0	161	436.0	14
**31**	1.63	7.42	21.7	228	177.0	22
**32**	1.71	8.72	30.9	146	549.6	8
**33**	1.70	8.18	27.6	101	414.4	6
**34**	1.67	7.80	24.6	270	64.5	>40
**35**	1.78	8.25	31.2	285	109.1	>40

**Table 7:** Physical and detonation properties of ionic salts of dinitropyrazoles. The data of compounds **36**–**39** are from reference [26], the data of compounds **40a**–**41o** and TATB are from reference [91].

Entry	*ρ*/g·cm^−3^	*D*/km·s^−1^	*P*/GPa	*T*_d_/°C	HOF/kJ· mol^−1^	IS/J	FS/N
**36**	1.69	-	-	127	-	40	360
**37**	1.70	8.11	25.9	300	-	40	360
**38**	1.63	7.59	21.1	156	-	40	360
**39**	1.59	7.32	19.1	295	-	40	360
**40a**	1.63	8.14	26.3	275	64.8	>60	-
**40b**	1.64	8.19	26.4	221	222.6	>60	-
**40c**	1.63	7.72	21.6	303	36.1	>60	-
**40d**	1.69	8.24	25.2	223	140.1	>60	-
**40e**	1.62	7.44	22.7	179	310.4	>60	-
**40f**	1.67	7.73	22.4	257	283.6	>60	-
**40g**	1.73	8.12	25.8	223	411.1	>60	-
**40h**	1.79	8.42	27.2	270	241.6	>60	-
**40i**	1.84	8.74	32.6	193	211.9	>60	-
**41j**	1.67	8.35	25.9	201	250.5	>60	-
**41k**	1.71	8.75	28.9	229	356.9	>60	-
**41l**	1.72	7.98	24.2	169	100.4	>60	-
**41m**	1.73	7.94	23.1	243	−166.3	>60	-
**41n**	1.54	7.71	21.0	206	389.3	>60	-
**41o**	1.60	7.78	22.4	173	471.8	>60	-
TATB	1.93	8.11	31.2	324	−140.0	50	-

**Table 8:** Physical and computational properties of ionic salts of dinitropyrazoles. The data of compounds **42a**–**43d** are from reference [94], the data of compounds **44**·H_2_O–**45** are from reference [95].

Entry	*ρ*/g·cm^−3^	*D*/km·s^−1^	*P*/GPa	*T*_d_/°C	IS/J	FS/N
**42a**	1.96	7.92	26.9	197	5	216
**42b**	1.68	8.28	28.2	167	10	360
**42c**	1.70	8.06	25.1	180	30	360
**42d**	1.64	8.02	24.7	169	10	360
**43a**	-	-	-	229	6	240
**43b**	1.62	7.91	24.4	224	30	288
**43c**	1.68	7.94	24.2	266	40	360
**43d**	1.68	8.16	25.7	131	10	360
**44**·H_2_O	1.86	-	-	93	20	240
**44a**	1.79	8.94	34.4	216	25	240
**44b**	1.86	9.00	37.6	182	35	360
**44c**	1.84	8.80	34.0	175	40	360
**44d**	1.71	8.20	26.4	204	40	360
**44e**	1.71	8.54	28.0	169	40	360
**44f**	175	8.88	30.7	214	40	360
**45**	1.80	8.81	33.9	212	40	360

**Table 9:** Physical and computational properties of several polynitropyrazoles. The data of compounds **46**–**52** are from reference [57], the data of compounds **53**–**54** are from reference [58].

Entry	*ρ*/g·cm^−3^	*D*/km·s^−1^	*P*/GPa	*T*_m_/°C	*T*_d_/°C	HOF/kJ·mol^−1^	IS/J	FS/N
**46**	1.71	7.46	20.1	58	241	200.3	>40	360
**47**	1.82	9.05	35.8	120	121	548.2	1.5	5
**48**	1.78	8.67	33.1	87	110	142.3	6	80
**49**	1.82	9.00	35.6	-	117	491.7	2.5	20
**50**	1.81	8.75	34.3	-	116	124.1	12	120
**51**	1.82	8.69	32.8	133	238.2	173.0	>40	360
**52**	1.83	8.80	35.0	-	134.4	112.0	8	80
**53**	1.91	8.67	35.5	80	157	244.0	8	130
**54**	1.94	8.73	36.6	81	159	206.0	9	145

**Table 10:** Physical and computational properties of **59**–**60i**. The data of compounds **59**–**59m** are from reference [101], the data of compounds **60**–**60i** and HMX are from reference [88].

Entry	*ρ*/g·cm^−3^	*D*/km·s^−1^	*P*/GPa	N/%	*T*_d_/°C	HOF/kJ·mol^−1^	IS/J	FS/N
**59**	1.84	9.17	37.8	44.2	270	833.4	9	240
**59a**	1.73	8.62	31.6	46.4	285	622.8	>40	>360
**59b**	1.76	8.83	34.4	44.0	215	709.9	33	252
**59c**	1.74	8.80	32.9	48.6	241	811.4	>40	>360
**59d**	1.78	8.66	31.1	48.8	340	624.1	>40	>360
**59e**	1.65	8.24	26.5	50.7	281	728.9	>40	>360
**59f**	1.70	8.54	28.9	52.4	262	831.7	27	240
**59g**	1.71	8.69	30.0	54.0	242	941.4	20	216
**59h**	1.72	8.36	28.8	51.0	279	828.3	>40	252
**59i**	1.74	8.56	29.5	52.6	292	944.8	>40	>360
**59j**	1.80	9.03	35.2	54.5	222	1211.7	12	252
**59k**	1.77	8.65	30.5	54.2	303	1166.1	20	>360
**59l**	1.75	-	-	41.0	261	-	7.5	252
**59m**	1.91	-	-	37.7	281	-	5	216
**60**	1.86	9.29	38.6	52.3	279	856.4	35	240
**60a**	1.79	8.95	33.3	54.3	299	672.6	>40	168
**60b**	1.84	9.23	37.4	51.1	296	719.7	>40	216
**60c**	1.84	9.36	37.0	56.4	290	819.8	>40	360
**60d**	1.67	8.26	25.9	56.0	256	648.8	>40	360
**60e**	1.72	8.76	28.6	61.2	216	808.7	>40	32
**60f**	1.79	9.07	32.5	59.4	285	844.9	>40	288
**60g**	1.81	9.29	34.2	60.9	287	954.9	>40	84
**60h**	1.84	8.95	32.2	57.6	286	840.2	>40	360
**60i**	1.82	9.00	32.3	59.1	261	960.0	>40	360
HMX	1.91	9.19	39.7	37.8	287	104.8	7.4	120

**Table 11:** Property parameters of salts of TNP. The data of compounds **61a**–**61m** are from reference [109], the data of compounds **62a**–**62d** are from reference [110], the data of compounds **63–63g** are from reference [111].

Entry	*ρ*/g·cm^−3^	*D*/km·s^−1^	*P*/GPa	*T*_d_/°C	HOF/kJ· mol^−1^	IS/J	FS/N
**61a**	1.73	8.46	29.9	224	60.5	40	-
**61b**	1.69	7.87	25.6	167	299.0	>40	-
**61c**	1.71	7.97	26.0	171	273.5	>40	-
**61d**	1.77	8.54	31.9	168	401.2	>40	-
**61e**	1.76	8.22	27.7	196	235.6	>40	-
**61f**	1.66	7.87	24.7	235	28.3	>40	-
**61g**	1.69	8.13	26.9	222	133.6	>40	-
**61h**	1.68	7.82	24.3	243	452.3	>40	-
**61i**	1.76	8.36	28.8	206	355.0	>40	-
**61j**	1.61	7.59	23.7	219	375.0	>40	-
**61k**	1.64	7.92	25.2	167	459.8	35	-
**61l**	1.62	7.98	25.3	197	246.5	>40	-
**61m**	1.65	8.24	27.2	184	352.7	>40	-
**62a**	-	-	-	274	-	40	96
**62b**	-	-	-	254	-	25	80
**62c**	-	-	-	193	-	40	80
**62d**	-	-	-	302	-	5	144
**63**	1.90	8.67	36.4	146	118.5	1	-
**63a**	1.82	8.68	35.1	176	35.1	6	-
**63b**	1.72	8.18	28.8	171	3.1	>40	-
**63c**	1.73	8.18	29.5	140	274.9	>40	-
**63d**	1.73	8.18	29.2	132	250.5	>40	-
**63e**	1.74	8.15	30.8	118	381.6	>40	-
**63f**	1.76	8.26	29.7	186	213.7	>40	-
**63g**	1.77	8.44	31.1	185	331.9	>40	-

**Table 12:** Physicochemical and energetic properties of compounds **64**–**76**. The data of compounds **64**–**67** are from reference [27], the data of compounds **70** are from reference [114], the data of compounds **71**–**73i** are from reference [116], the data of compounds **74**–**76** are from reference [117].

Entry	*ρ*/g·cm^−3^	*D*/km·s^−1^	*P*/GPa	*T*_m_/°C	*T*_d_/°C	HOF/kJ· mol^−1^	IS/J	FS/N
**64**	1.96	8.72	36.0	269	308	185.4	>40	-
**65**	1.89	8.60	35.0	dec	242	388.1	>40	-
**65a**	1.88	8.62	34.6	dec	262	274.7	>40	-
**65b**	1.73	8.04	27.3	dec	228	246.5	>40	-
**65c**	1.67	8.09	27.1	249	272	506.4	>40	-
**65d**	1.71	8.20	27.9	210	272	448.8	>40	-
**65e**	1.72	8.34	29.0	212	266	558.0	>40	-
**65f**	1.75	8.33	31.1	dec	259	331.2	>40	-
**65g**	1.82	8.45	31.0	247	297	557.0	>40	-
**65h**	1.72	8.23	28.9	166	261	700.4	>40	-
**65i**	1.80	8.54	32.8	dec	260	428.1	>40	-
**66**	1.82	8.81	37.0	158	297	824.2	28	-
**67**	1.87	8.65	35.1	260	284	477.9	>40	-
**70**	1.76	8.50	31.0	-	252	475.7	30	360
**71**	1.88	8.99	36.0	-	150	347.4	5	240
**72**	1.92	8.04	28.9	150	228	-50.7	6	120
**73a**	2.03	7.77	27.3	-	323	-125.2	4	40
**73b**	1.85	8.85	35.8	-	137	220.6	8	240
**73c**	1.77	8.67	31.5	94	155	220.9	10	240
**73d**	1.76	8.34	29.4	-	193	116.2	10	240
**73e**	1.69	8.14	25.2	-	196	353.3	15	360
**73f**	1.75	8.31	27.3	185	186	791.9	16	360
**73g**	1.76	8.22	26.5	-	206	565.4	12	360
**73h**	1.81	8.95	34.2	187	193	1359.4	10	360
**73i**	1.80	8.54	28.9	-	250	1269.7	18	360
**74**	1.79	7.53	22.1	377	382	203.5	30	>360
**75**	1.81	8.36	28.6	306	314	224.9	20	>360
**76**	1.86	8.52	31.1	292	298	227.8	4.5	192

**Table 13:** Physicochemical and energetic properties of **74**–**87**. The data of compounds **77–85** are from reference [122], the data of compounds **86**–**88**, CL-20 and DDNP are from reference [123], the data of compounds **89**–**90** are from reference [94], the data of compounds **91**–**92** are from reference [124].

Entry	*ρ*/g·cm^−3^	*D*/km·s^−1^	*P*/GPa	OB/%	*T*_d_/°C	HOF/kJ·mol^−1^	IS/J	FS/N
**77**	1.77	8.19	27.9	−17.2	311	218.9	>40	>360
**78**	1.84	8.75	34.3	3.5	80	380.6	7	80
**79**	1.72	7.80	24.2	−19.0	247	441.9	20	80
**80**	1.84	8.76	34.1	7.4	250	306.9	25	160
**81**	1.78	8.80	33.4	−7.5	112	1233.9	4	60
**82**	1.88	7.88	27.0	−7.8	319	230.0	>40	>360
**83**	1.76	8.56	31.0	−7.5	135	1013.9	3	60
**84**	1.75	8.13	27.3	−17.2	256	237.9	>40	>360
**85**	1.83	8.71	33.7	3.5	81	368.1	6	60
**86**	1.80	8.33	29.6	−40.2	310	205	11	>360
**87**	1.93	9.30	39.1	−11.5	205	379	4	144
**88**	1.73	8.02	26.0	−44.7	226	497	1.5	40
**89**	1.76	8.14	28.0	−39.0	319	302	25	360
**90**	1.72	7.97	26.3	−39.0	330	266	35	360
**91**	1.81	8.23	28.6	−39.0	262	224.2	14	352
**92**	1.81	8.36	29.7	−51.4	351	184.3	10	352
CL-20	2.04	9.67	44.9	−11.0	195	365	3	96
DDNP	1.72	76.5	23.8	−60.9	157	139	1	5

**Table 14:** Physical and energetic properties of energetic compounds **100**–**105**. The data of compounds **100**–**105** are from reference [127].

Entry	*ρ*/g·cm^−3^	*D*/km·s^−1^	*P*/GPa	*T*_d_/°C	HOF/kJ· mol^−1^	IS/J	FS/N
**100**	1.80	8.10	27.1	338	521.6	>40	>360
**101**	1.81	8.05	26.5	368	639.8	>40	>360
**102**	1.83	8.86	34.2	159	762.1	8	150
**103**	1.84	8.77	33.3	186	882.4	13	220
**104**	1.87	8.71	32.8	265	602.7	30	360
**105**	1.84	8.54	31.7	254	519.4	35	>360

**Table 15:** Physicochemical and energetic properties of compounds **107a**–**111**. The data of compounds **107a**–**111** are from reference [128].

Entry	*ρ*/g·cm^−3^	*D*/km·s^−1^	*P*/GPa	*T*_d_/°C	HOF/kJ· g^−1^	IS/J	FS/N
**107a**	1.90	8.79	34.3	261	1.10	15	240
**107b**	1.82	8.52	31.7	220	0.97	40	360
**108a**	1.86	8.89	35.9	221	1.05	35	360
**108b**	1.83	8.69	33.2	207	1.33	25	360
**109**	1.79	8.36	29.6	242	0.96	15	160
**110**	1.94	9.23	38.8	117	1.30	3	20
**111**	1.87	9.03	37.1	138	1.32	10	80

**Table 16:** Properties of nitrated bispyrazoles from 1,3-dichloro-2-nitro-2-azapropane. The data of compounds **115**–**122** are from reference [130].

Entry	*ρ*/g·cm^−3^	*D*/km·s^−1^	*P*/GPa	*T*_d_/°C	HOF/kJ· mol^−1^	IS/J	OB/%
**115**	1.69	7.87	25.1	262	377.2	>40	−30.8
**116**	1.78	8.26	30.9	250	388.0	10	−4.0
**117**	1.78	8.27	31.0	261	398.0	>40	−4.0
**118**	1.90	8.06	30.6	252	371.8	11	0
**119**	1.86	8.64	34.7	232	486.4	>40	−7.4
**120**	1.89	8.04	30.4	354	381.3	>40	0
**121**	1.83	8.72	35.1	166	1108.2	2	0
**122**	1.83	8.72	35.2	169	1118.7	2	0

**Table 17:** Properties of energetic compounds **123a**–**m**. The data of compounds **123a**–**123m** are from reference [138].

Entry	*ρ*/g·cm^−3^	*D*/km·s^−1^	*P*/GPa	*T*_d_/°C	HOF/kJ· mol^−1^	IS/J	FS/N	OB/%
**123a**	1.69	8.21	25.4	328	158.5	>40	360	−27
**123b**	1.82	9.01	35.4	327	274.2	29	360	−12
**123c**	1.72	8.86	30.3	247	501.0	16	160	−30
**123d**	1.71	8.04	24.5	287	481.9	>40	360	−30
**123e**	1.67	8.23	24.6	289	963.8	>40	360	−41
**123f**	1.68	7.95	22.5	324	173.3	>40	360	−40
**123g**	1.69	8.40	25.6	222	477.0	>40	360	−41
**123h**	1.71	8.73	28.0	209	679.6	>40	360	−42
**123i**	1.76	8.81	29.9	215	605.5	12	80	−31
**123j**	1.79	8.36	27.9	238	505.6	23	160	−27
**123k**	2.14	-	-	395	-			0
**123l**	2.20	-	-	365	-			0
**123m**	3.27	-	-	327	-			0

**Table 18:** Physical and detonation properties of energetic compounds LLM-119 and **124**–**130**. The data of compounds LLM-119 and **124**–**126i** are from reference [141], the data of compounds **127**–**130** are from reference [142].

Entry	*ρ*/g·cm^−3^	*D*/km·s^−1^	*P*/GPa	*T*_d_/°C	HOF/kJ·mol^−1^	IS/J	FS/N	OB/%	N + O/%	I_sp_/s
**LLM-119**	1.84	8.86	33.9	230	467.0	15	160	−14.0	77.2	246
**124**	1.82	8.67	33.1	206	133.7	10	120	9.2	78.2	245
**125**	1.96	9.46	40.9	145	550.9	3	20	22.2	83.3	269
**126**	1.93	9.51	41.8	128	595.2	2	20	15.1	84.3	274
**126a**	1.81	8.98	35.9	181	423.1	10	120	0	84.1	270
**126b**	1.85	9.40	39.5	174	738.9	5	60	−4.2	84.8	280
**126c**	1.88	9.50	41.3	170	531.2	7	120	8.3	85.4	282
**126d**	1.68	8.30	26.9	190	454.7	35	360	−14.7	80.7	239
**126e**	1.71	8.61	29.3	153	692.9	30	360	−17.2	81.5	247
**126f**	1.70	8.88	30.8	141	1144.8	10	80	−21.3	82.8	258
**126g**	1.78	9.17	36.0	163	1683.3	5	60	−9.3	84.2	280
**126h**	1.83	9.00	33.1	203	1599.5	10	120	−23.0	78.6	244
**126i**	2.11	8.31	31.2	208	152.9	2	20	16.2	68.0	226
**127**	1.74	7.93	27.9	178	356.0	14	280	−41.3	76.0	-
**128**	1.83	8.48	32.8	208	18.8	12	160	−18.4	78.2	-
**129**	1.74	7.82	27.1	198	863.0	10	240	−51.9	75.3	-
**130**	1.90	8.84	36.5	296	269.0	16	300	−20.9	78.2	-

**Table 19:** Physicochemical and energetic properties of **133** and its ionic salts. The data of compounds **133**–**133f** are from reference [146].

Entry	*ρ*/g·cm^−3^	*D*/km·s^−1^	*P*/GPa	*T*_d_/°C	HOF/kJ·mol^−1^	IS/J	FS/N
**133**	1.79	8.72	30.9	281	1111.5	15	192
**133a**	2.00	8.81	28.5	329	638.9	25	252
**133b**	1.69	8.40	26.2	280	916.8	19	>360
**133c**	1.61	8.24	26.0	178	1062.2	27.5	324
**133d**	1.75	9.08	31.3	221	1223.0	12	144
**133e**	1.62	8.02	22.4	299	926.9	>60	>360
**133f**	1.64	8.40	24.9	255	1143.3	35	>360

## Caution

Readers are reminded that the information given in this review is intended to cover the progress of recent research on energetic azo materials. Most of the molecules collected in this review are energetic materials that may be explosive under certain conditions. Their syntheses should be carried out by experienced personnel and handled with caution. In any case, carefully planned safety protocols and proper protective equipment, such as Kevlar gloves, ear protection, safety shoes and plastic spatulas, should be utilized at all times, especially when working on a large scale (>1 g). The authors strongly suggest that the original references be consulted for detailed safety information.
